# Predicting academic success of autistic students in higher education

**DOI:** 10.1177/13623613221146439

**Published:** 2023-01-05

**Authors:** Theo Bakker, Lydia Krabbendam, Sandjai Bhulai, Martijn Meeter, Sander Begeer

**Affiliations:** Vrije Universiteit Amsterdam, The Netherlands

**Keywords:** academic achievement, autism, higher education, predictive modeling, propensity score weighting, student retention

## Abstract

Individuals with autism increasingly enroll in universities, but little is known about predictors for their success. This study developed predictive models for the academic success of autistic bachelor students (*N* = 101) in comparison to students with other health conditions (*N* = 2465) and students with no health conditions (*N* = 25,077). We applied propensity score weighting to balance outcomes. The research showed that autistic students’ academic success was predictable, and these predictions were more accurate than predictions of their peers’ success. For first-year success, study choice issues were the most important predictors (parallel program and application timing). Issues with participation in pre-education (missingness of grades in pre-educational records) and delays at the beginning of autistic students’ studies (reflected in age) were the most influential predictors for the second-year success and delays in the second and final year of their bachelor’s program. In addition, academic performance (average grades) was the strongest predictor for degree completion in 3 years. These insights can enable universities to develop tailored support for autistic students. Using early warning signals from administrative data, institutions can lower dropout risk and increase degree completion for autistic students.

**Laymen Summary:**

**What is already known about the topic?**

Autistic youths increasingly enter universities. We know from existing research that autistic students are at risk of dropping out or studying delays. Using machine learning and historical information of students, researchers can predict the academic success of bachelor students. However, we know little about what kind of information can predict whether autistic students will succeed in their studies and how accurate these predictions will be.

**What does this article add?**

In this research, we developed predictive models for the academic success of 101 autistic bachelor students. We compared these models to 2,465 students with other health conditions and 25,077 students without health conditions. The research showed that the academic success of autistic students was predictable. Moreover, these predictions were more precise than predictions of the success of students without autism.

For the success of the first bachelor year, concerns with aptitude and study choice were the most important predictors. Participation in pre-education and delays at the beginning of autistic students’ studies were the most influential predictors for second-year success and delays in the second and final year of their bachelor’s program. In addition, academic performance in high school was the strongest predictor for degree completion in 3 years.

**Implications for practice, research, or policy**

These insights can enable universities to develop tailored support for autistic students. Using early warning signals from administrative data, institutions can lower dropout risk and increase degree completion for autistic students.

## Introduction

Autism (Autism Spectrum Disorder; ASD; American Psychiatric Association [APA], 2013) is a neurodevelopmental condition characterized by qualitative differences in social interaction, communication, repetitive, stereotyped behavior, and sensory perception. As the number of autistic students in higher education increases (Bakker et al., 2019; Nuske, Rillotta, et al., 2019; Zeedyk et al., 2016), insights into their academic success grow (Anderson et al., 2017). Innovative statistical analyses, such as machine learning to predict autistic students’ academic success, could enhance our understanding, but data-driven research is still limited (Chown et al., 2016). To uncover differences in academic success patterns between autistic students and their peers in this preregistered study (Bakker et al., 2021), we studied the feasibility of predictive modeling of academic success and the importance of success predictors. We define academic success as the completion of a bachelor’s degree; the opposite, dropping out, indicates a lack of academic success.

Predictive modeling is a machine learning technique to predict future observations and evaluate their predictive power. Predictive modeling plays a vital role in theory building. It helps to uncover new predictive measures, create new hypotheses, find improvements to existing explanatory models, compare competing theories, and quantify predictive accuracy (Shmueli & Koppius, 2011). Predictive modeling in higher education (Namoun & Alshanqiti, 2020) can elucidate educational outcomes, evaluate policies, and help to apply interventions (Jia & Maloney, 2015). Autistic students risk delaying or dropping out (Chown et al., 2016; Van Hees et al., 2015). Their transition to university is challenging, as they leave familiar support and routines and face new relationships and academic demands (Beardon et al., 2009; Lambe et al., 2018). Evidence-based warning systems are necessary to detect at-risk autistic students and offer them support (Anderson et al., 2019; Gelbar et al., 2014; Meinel et al., 2021; Zeedyk et al., 2016).

The number of studies on the application of predictive algorithms in the field of autism is growing (Hyde et al., 2019; Thabtah, 2018; Vabalas et al., 2019), in particular related to diagnostic or neurological aspects. Their use in educational studies is scarce (Madaus et al., 2020; Morgan, 2018). Qualitative literature identified factors influencing autistic academic success (Accardo et al., 2019; Cage et al., 2020; Cox et al., 2021; Francis et al., 2017; Gillespie-Lynch et al., 2017; Shmulsky et al., 2017). Several factors were related to the student, such as autism-related characteristics (high intrinsic motivations, attention to detail, intense interests, executive functioning and social skills, inflexibility, sensory overstimulation, and mental health), identity (self-awareness, self-advocacy, and achieving goals), and connections (family support, relationships with and understanding from professors and peers). Other factors were related to the institution, such as norms (accommodation requirements, good grades), formal accommodations (transition planning, financial aid, extended time or distraction-free environment), and personalized adaptations. No studies employ predictive modeling to predict autistic students’ academic outcomes or compare autistic and nonautistic students.

Predictive modeling in higher education aims to make successful predictions to improve student outcomes (Alyahyan & Düştegör, 2020). About 70% of studies on the prediction of higher education success predict factors of study progression, such as student grades. In comparison, only 10% predict longitudinal student outcomes, that is, dropping out or completing a degree (Hellas et al., 2018). To develop timely interventions for autistic students, early and effortless predictions of longitudinal student outcomes are needed. Accordingly, it is crucial to select a limited set of the most influential features available the student begins their academic studies, such as background characteristics (sex, age, pre-education), earlier educational outcomes (grades), and motivation (Bonifro et al., 2020; Jia & Maloney, 2015; Khan & Ghosh, 2021; Thabtah, 2018).

We studied longitudinal, balanced data of a comprehensive population sample across three groups at a major Dutch university (*N* = 27,643): autistic students (AS); students with other conditions (OC), including ADD/ADHD and dyslexia; and students with no conditions (NC). We compared autistic and nonautistic students to predict their academic success after each bachelor year. We developed five predictive models based on seven cohorts of historical student data. We selected and studied features that were available before the beginning of their studies and are registered and readily available in most administrative systems of higher education institutions. We expect predictive modeling of autistic students’ academic success to be feasible and show different important predictors compared to their peers.

## Methods

### Study population

Our convenience sample included 27,643 first-year, full-time students in 54 bachelor programs of the Vrije Universiteit Amsterdam, from 2010 to 2016 (M = 19 years old, 55.0% female) with study measures from the student information system of the university. This research university offers a range of full-time bachelor and master programs in Humanities, Natural sciences, Social sciences & Law, and Health & Life sciences. It attracts students from the greater area of Amsterdam and abroad. The Dutch higher education system follows the European Bologna Process, which includes 48 European countries (European Commission, n.d.), with a three-cycle higher education system consisting of bachelor’s, master’s, and doctoral studies, mutual recognition of qualifications, and a common system of quality assurance.

The university created the data set from its student information systems, providing validated, uniform, and anonymized student data. Specific data on socioeconomic status and ethnicity were not recorded. The institution’s Scientific and Ethical Review Board granted ethical clearance (reference number VCWE-2017-123).

The study population consisted of three participant groups: (1) 101 students with a clinical diagnosis of ASD (0.37%; comorbidity: 28.7%), (2) 2,465 students with other conditions (8.92%; ADD/ADHD: 1.36%; chronic diseases: 1.02%; dyslexia: 4.43%; physical disabilities: 0.96%; psychological disabilities: 0.54%; and other disabilities, such as language deficiency and deafness: 1.25%; comorbidity: 11.6%), and (3) 25,077 students with no recorded conditions (90.72%). The prevalence of autism in the Netherlands is comparable to other countries (Hoekstra, 2018). However, the prevalence of autistic students and students with other conditions is low. As autistic students in this study were restricted to those who disclosed their diagnosis, we expect the actual prevalence of autistic students to be more in agreement with estimations between 1.0% and 1.5% (Centers for Disease Control and Prevention, 2022). Autistic students, and students with other conditions, included only those who disclosed their formally registered diagnosis (Bakker et al., 2020) provided by qualified clinicians independently from this study. We collapsed the six non-ASD disability categories into one group.

One-third of autistic students in the Netherlands report barriers in their studies, but many students with other disabilities or without disabilities face similar issues (Van den Broek et al., 2017; Van Rooij et al., 2018). About 30% of the total student population has a disability; one-third of students with disabilities experience many impediments in their studies (10% to 11%; Van den Broek et al., 2013).

In the Netherlands, a psychiatrist diagnoses ASD according to the established Diagnostic and Statistical Manual of Mental Disorders (4th ed., text rev.; *DSM*-IV-TR) or Diagnostic and Statistical Manual of Mental Disorders (5th ed.; *DSM*-5) criteria based on a detailed examination, including observations and parent interviews by multiple experienced clinicians (psychologists, psychiatrists, and educators).

### Measures

See Appendix Table 5 for a list of all variables, their measurement scales, and their application in propensity score weighting (PSW) or predictive modeling (PM).

#### Demographic and enrollment characteristics

*Sex* is male or female. *Age (in years)* in Dutch higher education is recorded on October 1 in the year students enroll. *Cohort* is the academic year a student enrolled for the first time in their academic program (Bakker et al., 2019). *Days between application and September 1* is the number of days between the application and the start of the first bachelor year (September 1). *Parallel program* indicates whether a student enrolled in one or more other bachelor programs in the first bachelor year. *STEM* shows whether a student enrolled in a science, technology, engineering, and mathematics study program, based on the STEM Designated Degree Program List (2016).

#### Educational background

*Regular pre-education*. In the Netherlands, there are five learning paths to higher education: (a) high school VWO (Voorbereidend Wetenschappelijk Onderwijs, university preparatory education), (b) a vocational foundation year (high school HAVO (Hoger Algemeen Voortgezet Onderwijs, senior general secondary education) with the first-year qualification from a university of applied sciences), (c) a qualification in Dutch higher education (academic or vocational), (d) other Dutch qualifications, such as a university entrance examination (colloquium doctum), and (e) a foreign qualification equivalent to VWO (Bakker et al., 2020). We classified regular pre-education as true if the highest pre-education was not classified as (d) other Dutch qualifications. *Average grade secondary education* is the average grade of all subjects a student chose to graduate in; grades range from 1 to 10. *Average grade secondary education missing* is an indication if the average grade secondary education is missing. *Average grade math secondary education* is the average grade in algebra in secondary school; grades range from 1 to 10. *Average grade math secondary education missing* is an indication if the average grade *math* secondary education is missing.

#### Success

All bachelor programs consist of 180 European Credits (ECs) with 60 ECs in three academic years. *Dropout after 1* *year* and *Dropout after 2* *years* means that a student did not enroll in the same study program in the following academic year. We derived *success after 3* *years* from dropout after 1, 2, or 3 years, and degree completion within 3 years. If a student dropped out within three academic years, they were categorized as a “dropout”; if a student received a degree after 3 academic years, they were categorized as “degree”; and otherwise, they were categorized as “re-enrolled.”

### Analytical strategy

We used R for statistical computing, version 4.1.0, for data wrangling and analysis (R Core Team, 2017). We analyzed the outcomes using PSW to address biases associated with the differences in group sizes. The propensity score is a number between zero and one and represents the conditional probability that a person is assigned to a particular group, given a set of confounders (Austin, 2011). We assessed covariate balance using the cobalt package, version 4.2.3 (Greifer, 2019). We predicted academic success after 3 years using predictive modeling (Alyahyan & Düştegör, 2020).

#### Data selection, imputation, propensity score weighting, and variable balance evaluation

We imputed the dataset to prevent bias associated with list-wise deletion. We did not impute values for the outcomes or disability (Newman et al., 2018). The measures sex, highest pre-education, cohort, and average grade math secondary education with median imputation and stop method maximum absolute standardized mean difference (es. max) gave the best balance, with an overlap in the interquartile range of 10.9% for AS-OC and 14.3% for AS-NC. We kept the sample size of AS constant to 101 and reduced the sample size of OC from 2465 to a weighted size of 89.38 and NC from 25,077 to a weighted size of 92.28. Table 1 presents the balance of AS, OC, and NC. The weighted samples represent the best-matched comparison between the three groups.

**Table 1.** Balance of the treatment and comparison groups without outliers.

Measures	Unweighted means (%)			Weighted means (%)			Population
	AS	OC	NC	AS	OC	NC	Mean (%)
Sex							
Male	0.71	0.41	0.45	0.71	0.62	0.61	0.45
Female	0.29	0.59	0.55	0.29	0.38	0.39	0.56
Highest pre-education							
High school VWO	0.75	0.80	0.83	0.75	0.81	0.81	0.83
Vocational foundation year	0.13	0.14	0.10	0.13	0.12	0.09	0.10
Degree in higher education	0.02	0.04	0.05	0.02	0.03	0.04	0.04
Other Dutch pre-education	0.10	0.03	0.02	0.10	0.04	0.05	0.02
Cohort							
2010	0.11	0.10	0.19	0.11	0.09	0.15	0.18
2011	0.21	0.12	0.16	0.21	0.16	0.18	0.16
2012	0.11	0.13	0.14	0.11	0.12	0.12	0.14
2013	0.07	0.15	0.14	0.07	0.13	0.11	0.14
2014	0.20	0.18	0.13	0.20	0.18	0.17	0.14
2015	0.17	0.17	0.11	0.17	0.17	0.13	0.12
2016	0.14	0.16	0.13	0.14	0.15	0.14	0.12
Average grade							
Average grade math *SE*	6.54	6.59	6.57	6.54	6.52	6.52	6.55
Not missing	0.85	0.92	0.93	0.85	0.94	0.93	0.94
Missing	0.15	0.08	0.07	0.15	0.06	0.07	0.06

AS: autistic students; OC: students with other conditions; NC: students with no recorded conditions; SE: secondary education; VWO: Voorbereidend Wetenschappelijk Onderwijs.

#### Transformation and outlier removal

As none of the continuous measures were normally distributed, we transformed the data using the best Normalize package, version 1.8.0 (Peterson & Cavanaugh, 2019). We used centering and scaling for age and days between the application and September 1, log10 transformation for average grade secondary education, and square root transformation for average grade math algebra secondary education. For optimal model convergence, we scaled continuous measures to a range between 0 and 1. We remove outliers that were more than three standard deviations (z-scores) away from the mean (AS: 4, 3.96%; OC: 69, 2.80%; NC: 893, 3.56%; weighted totals: AS: 97.0, OC: 91.1, NC: 90.3; unweighted totals: AS: 97, OC: 2,396, NC: 24,184). Table 2 presents the descriptive statistics of AS, OC, and NC without outliers.

**Table 2.** Background, enrollment, and success characteristics for the three participant groups (*N* = 26,677).

	AS	OC	NC	*p* value	Group differences
	*N* = 97	*N* = 2396	*N* = 24,184		
Age (in years)	19.0 [18.0–21.0]	19.0 [18.0–21.0]	19.0 [18.0–20.0]	<0.001	AS *>* NC; OC *>* NC
Sex: Female	27 (27.8%)	1,414 (59.0%)	13,352 (55.2%)	<0.001	AS *<* OC and NC
Average grade SE	6.7 [6.4–7.1]	6.6 [6.3–6.9]	6.6 [6.3–6.9]	0.024	OC *<* NC
Average grade math SE	6.0 [6.0–7.0]	6.5 [6.0–7.0]	6.5 [6.0–7.0]	1.000	n.s.
Regular pre-education: True	87 (89.7%)	2333 (97.4%)	23,679 (97.9%)	<0.001	AS *<* OC and NC
Cohort:				<0.001	
2010	11 (11.3%)	244 (10.2%)	4,648 (19.2%)		AS *>* OC; AS *<* NC
2011	20 (20.6%)	294 (12.3%)	3,928 (16.2%)		AS *>* NC *>* OC
2012	11 (11.3%)	301 (12.6%)	3,447 (14.3%)		AS *<* OC *<* NC
2013	7 (7.2%)	361 (15.1%)	3,490 (14.4%)		AS *<* NC *<* OC
2014	19 (19.6%)	424 (17.7%)	3,224 (13.3%)		AS *>* OC *>* NC
2015	16 (16.5%)	403 (16.8%)	2,697 (11.2%)		AS *<* OC; AS *>* NC
2016	13 (13.4%)	369 (15.4%)	2,750 (11.4%)		AS *<* OC; AS *>* NC
Days between application and September 1	129.0 [69.0-–168.0]	128.0 [64.0–176.0]	125.0 [56.0–168.0]	<0.001	OC *>* NC
STEM	51 (52.6%)	739 (30.8%)	6,794 (28.1%)	<0.001	AS *>* OC and NC
Parallel program: True	4 (4.1%)	84 (3.5%)	744 (3.1%)	1.000	n.s.
Dropout after 1 year	23 (23.7%)	492 (20.5%)	6,533 (27.0%)	<0.001	OC *<* NC
Dropout after 2 years	36 (37.1%)	614 (25.6%)	7,613 (31.5%)	<0.001	AS *>* OC; OC *<* NC
Success after 3 years				<0.001	
Degree	20 (20.6%)	584 (24.4%)	7,226 (29.9%)		AS *<* OC *<* NC
Dropout	36 (37.1%)	662 (27.6%)	7,928 (32.8%)		AS *>* NC *>* OC
Re-enrolled	41 (42.3%)	1,150 (48.0%)	9,030 (37.3%)		AS *>* OC; AS *<* NC

AS: students with ASD; NC: students with no recorded conditions; n.s.: no significant group differences; OC: students with other conditions; SE: secondary education.

#### Predictive modeling

We selected features that were available before the first enrollment (Bonifro et al., 2020; Chiang et al., 2012; Jia & Maloney, 2015) and are generally used in predictive modeling in higher education (Khan & Ghosh, 2021; Pingry O’Neill et al., 2012; Van Rooij et al., 2018): demographics (sex, age), educational background (type of pre-education), previous outcomes (average grade in secondary education and average grade math in secondary education), and the field of study (science, technology, engineering, and math [STEM] both true and false). We included two additional measures that could be correlated to academic success or delays: (a) days between the application and September 1 as an indicator of motivation, executive functioning, and procrastination (Anderson, 2018; Nuske, Rillotta et al., 2019a; Robertson & Ne’eman, 2008; Van Hees et al., 2015; Vincent, 2019) and (b) parallel program, because following two study programs heightens the risk of dropping out. The final model formula is outcome measure ∼ sex + age + regular pre-education + average grade secondary education + average grade math secondary education + average grade secondary education (missing) + average grade math secondary education (missing) + regular pre-education + days between application and September 1 + STEM + parallel program.

To perform the steps in the predictive modeling process (Shmueli, 2010), we used the caret package, version 6.0.88 (Kuhn, 2021), for partitioning of the data; selection of important predictors, that is, features; building the prediction models; validation and evaluation of the models; and selection of the optimal model. To avoid imbalance bias, we created stratified, balanced data splits for each research group (Meinel et al., 2021). Random sampling occurred within each class of the dependent measures and preserved the overall class distribution of the data (Hellas et al., 2018). To prevent bias in accuracy for small samples, a possible risk in machine learning research in the field of autism (Vabalas et al., 2019), and to reduce the risk of overfitting for larger samples (Leppink, 2020), we applied 10 × 10-fold repeated cross-validation as the resampling scheme. We created the train and test sets in three splits within each research group (80/20%, 70/30%, and 60/40%).

To predict the outcomes and assess variable importance, we built and trained five weighted models that cover various possibilities in predictive modeling for multiclass outcomes: classification and regression trees, random forest, neural network, boosting, and bagging models. *Classification and regression trees* are obtained by recursively partitioning data and fitting a simple prediction model within each partition. The partitioning can be represented as a decision tree. Classification trees are designed for dependent variables that take a finite number of unordered values, with prediction error measured in misclassification cost (Loh, 2011). *Random forest* combines several randomized decision trees and aggregates their predictions by averaging (Biau & Scornet, 2016). *Penalized multinomial regression* generalizes logistic regression to multiclass problems, utilizing penalties to improve the fit of the data (Greene, 2012; Kuhn & Johnson, 2013). *Stochastic gradient boosting* constructs additive regression models by sequentially fitting a regression tree (base learner) by least squares. At each iteration, a fraction of the training data is drawn at random without replacement to fit the base learner (Friedman, 2002). *Bagged classification and regression trees* aggregate bootstrap samples of the original data to reduce the variance of classification and regression trees (Breiman, 1996).

We analyzed (1) classification and regression trees (importing the rpart package, version 4.1.15 [Therneau & Atkinson, 2019]); (2) random forest (importing ranger, version 0.12.1 [Wright & Ziegler, 2017]); (3) penalized multinomial regression (importing nnet, version 7.3.16 [Venables & Ripley, 2002]); (4) stochastic gradient boosting (importing gbm, version 2.1.8 [Greenwell et al., 2020]); (5) bagged classification and regression trees (importing ipred, version 0.9.11 [Peters & Hothorn, 2021]). To reduce the data dimensionality of relevant features (Rahman et al., 2020), we determined the variable importance for each model based on the training set using the packages’ built-in methods.

We compared the performance of the models on the training and test sets with confusion matrices using classification accuracy with 95% confidence intervals (CIs), *κ*, and the no information rate (NIR). We applied a one-sided binomial test. The NIR is the best guess given no information beyond the overall distribution of the prediction classes, which is the largest class percentage of the outcome measure, that is, the majority class (Kuhn, 2015). A predictive model should at least outperform the NIR to add predictive value.

We tested all models with a random selection of 97 OCs and 97 NCs with the same cohort distribution as AS to exclude misinterpretations due to differences in sample size.

### Preregistration

Following this study’s preregistration (Bakker et al., 2021), we report additional data exclusions, inclusions, and changes. To contrast predictive models on academic success over the years, we added predictive models for dropouts after 1 and 2 years. Because one of the predicted outcomes was categorical with three levels, we applied appropriate predictive modeling procedures. We selected models that accepted case weights and represented different types of models.

### Community statement

As nonautistic autism researchers, we value community involvement and are motivated to compare students with and without autism. Having been nonautistic students might have biased us by viewing neurotypical normality in higher education, such as enjoying social life or extracurricular activities, as the norm. This study was part of a Ph.D. project that was discussed in the community of researchers of the Netherlands Autism Register (NAR), with strong involvement of autistic stakeholders.

## Results

### Prediction models

For AS, the best-performing models are penalized multinomial regression for dropout after 1 year, and random forest for dropout after 2 years and success after 3 years. There appears to be no optimal technique per group or success measure. See Table 3 for the performance metrics of each group’s best-performing models.

**Table 3.** Best performing models for the three participant groups.

Outcome	Group	Model	Split	Set	Accuracy (95% CI)	*κ*	NIR (%)	*p* value
Dropout	AS	PMR	80/20%	Test	83.3% (58.6%–96.4%)	0.34	77.8	0.409
After 1 year				Train	77.2% (66.4%–85.9%)	0.12	75.9	0.457
	OC	SGB	60/60%	Test	79.7% (77.0%–82.2%)	0.02	79.5	0.455
				Train	79.5% (77.3%–81.6%)	0.01	79.4	0.490
	NC	SGB	70/30%	Test	73.2% (72.1%–74.2%)	0.02	73.0	0.381
				Train	73.0% (72.3%–73.6%)	0.01	73.0	0.518
Dropout	AS	RF	70/30%	Test	75.0% (55.1%–89.3%)	0.39	64.3	0.162
After 2 years				Train	73.9% (61.9%–83.7%)	0.36	62.3	0.029*
	OC	PMR	80/20%	Test	74.7% (70.5%–78.5%)	0.04	74.5	0.482
				Train	74.8% (72.8%–76.7%)	0.04	74.3	0.349
	NC	SGB	70/30%	Test	69.9% (68.9%–71.0%)	0.15	68.5	0.005**
				Train	70.1% (69.4%–70.8%)	0.15	68.5	<0.001***
Success	AS	RF	70/30%	Test	64.3% (44.1%–81.4%)	0.40	42.9	0.018*
After 3 years				Train	68.1% (55.8%–78.8%)	0.47	42.0	<0.001***
	OC	PMR	70/30%	Test	51.1% (47.4%–54.8%)	0.13	48.1	0.054 .
				Train	50.4% (47.9%–52.8%)	0.12	48.0	0.027*
	NC	RF	60/60%	Test	47.0% (46.0%–48.0%)	0.20	37.3	<0.001***
				Train	60.8% (60.0%–61.6%)	0.41	37.3	<0.001***

AS: students with ASD; CI: confidence Interval; NC: students with no recorded conditions; NIR: no information rate; OC: students with other conditions; RF: random forest; PMR: penalized multinomial regression; SGB: stochastic gradient boosting; . = *p* < 0.1, *p < 0.05, **p < 0.01, ***p < 0.001.

The improved models outperformed the simple classification and regression tree models. The predictive power of all models was low on both the training and test sets. None of the models was significant, except for the NC model for dropout after 2 years and success after 3 years. However, the best-performing model for each outcome and group outperformed the NIR, that is, the best guess based on the majority class. The benchmark of the models’ accuracy shows that the predictability of academic success is higher for autistic students than for their peers. This difference increases throughout the bachelor program as the ratio between the prediction accuracy and the NIR increases for AS (dropout after 1 year: 5.5%, OC: 0.2%, NC: 0.2%; dropout after 2 years: AS: 10.7%, OC: 0.2%, NC: 1.4%; success after 3 years: AS: 21.4%, OC: 3.0%, NC: 9.7%). See Supplement 1 for the performance metrics of all models.

### Feature selection and variable importance

See Table 4 for the variable importance of each group’s best-performing models for each outcome.

**Table 4.** Variable importance.

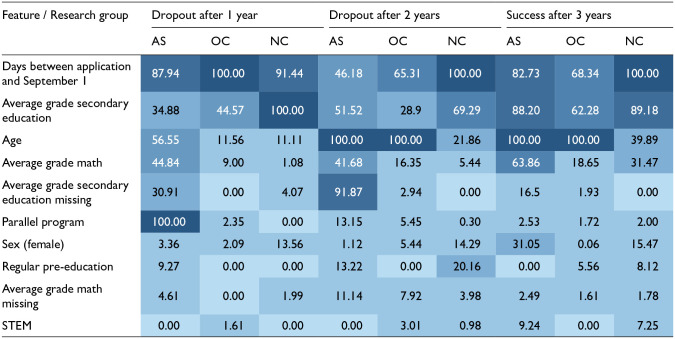

AS: students with ASD; NC: students with no recorded conditions; OC: students with other conditions; features > 40.00 printed in white.

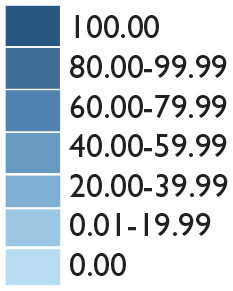

The model’s feature selection and variable importance were different for each group for all outcome measures. The number of important features (importance > 40%) for the AS models was higher than for the OC and NC models and different in rank (dropout after 1 year: AS: 4 features (Parallel program: 100.00; Days between application and September 1: 87.94; Age: 56.55; Average grade math: 44.84), OC: 2 features (Days between application and September 1: 100.00; Average grade secondary education: 44.57), NC: 2 features (Average grade secondary education: 100.00; Days between application and September 1: 91.44); dropout after 2 years: AS: 5 features (Age: 100.00; Average grade secondary education missing: 91.87; Average grade secondary education: 51.52; Days between application and September 1: 46.18; Average grade math secondary education: 41.68), OC: 2 features (Age: 100.00; Days between application and September 1: 65.31), NC: 2 features (Days between application and September 1: 100.00; Average grade secondary education: 69.29); success after 3 years: AS: 4 features (Age: 100.00; Average grade secondary education: 88.20; Days between application and September 1: 82.73; Average grade math secondary education: 63.86), OC: 3 features (Age: 100.00; Days between application and September 1: 68.34; Average grade secondary education: 62.28), NC: 2 features (Days between application and September 1: 100.00; Average grade secondary education: 89.18)).

## Discussion

This longitudinal study examined (1) whether it is possible to predict academic success after three bachelor years and (2) which aspects are important predictors. Predicting the academic success of autistic students was feasible. The best-performing models for the academic success of autistic students outperformed the NIR models for dropout after 1 and 2 years and academic success after 3 years. Over the years of the bachelor program, this difference in performance increases. Furthermore, the study shows that the academic success of autistic students is more predictable than their peers’. The accuracy of autistic students’ success models is higher than their peers, success models. In addition, the differences between the accuracy of autistic students’ best-performing models and the NIR models are more extensive than those for students without autism. A reason could be that the variation in autistic students is smaller than in students with other conditions or no conditions, which gives predictions more power.

The differences in order and importance of the predictors for the three groups provide more insight into the underlying mechanisms that could influence academic success over the three bachelor years. Out of the ten predictors, three appear to be the strongest for all students (>40%): (1) age, (2) the average grade in secondary education, and (3) the number of days between their application and the start of the academic year. For autistic students, additional predictors are (4) parallel program, (5) average grade math in secondary education, and (6) average grade secondary education (missing). See Table 4 for a comparison of variable importance and patterns.

The variable importance of predictors in machine learning can reveal underlying mechanisms for theory building (Shmueli & Koppius, 2011). In the following sections, we will discuss the prediction of the academic success of autistic students in three bachelor years. We will explain how the most important predictors are proxies for insights into three mechanisms for their academic success: (1) study choice and transition to higher education (the number of days between their application and the start of the academic year, parallel program), (2) long-term issues with earlier educational fit and inclusion (age, average grade secondary education (missing), and (3) earlier academic performance (average grade in secondary education, average grade math in secondary education).

### Prediction of first-year dropout

Enrollment in a parallel program and the number of days between application and the start of the academic year are the most important predictors of autistic students’ first-year success. These predictors reveal information on study choice and transition to higher education. Only 4.1% of autistic students enrolled in parallel bachelor programs. They enrolled earlier than their peers (129 days before September 1, 95% CI: 69–168). The second field of study could be a sign of strong academic commitment or the opposite: uncertainty and postponement of the choice of a field of study (Van den Broek et al., 2017). Reasons for dropping out of a parallel program are heavy study load (Ward & Webster, 2018), transition barriers (Anderson, 2018; White et al., 2017; Zeedyk et al., 2019), or time management and planning (Nuske, McGhee Hassrick, et al., 2019). The number of days is a proxy for short-term problems that delay applications and captures personal or academic issues, which could influence a later tendency to dropout or delay (Jansen et al., 2016; Van den Broek et al., 2013; Van den Broek et al., 2017; Van Rooij et al., 2018). Students who applied later might have had issues in high school examinations, resits, choosing their program, or switching from other programs. An additional explanation might be that autistic students or their parents received less transition planning support.

Prediction of the first-year dropout shows that autistic students at risk of dropping out have difficulties transitioning to the university. The relatively late application demonstrates uncertainty, or their study choice for an additional program is less viable than autistic students who persist.

### Prediction of second-year dropout

In the second year, the order of predictors reverses. Dropout in the first bachelor year has taken its toll on almost one in four autistic students (23.7%). As a result, more profound issues with participation in pre-education have surfaced that influence second-year dropout rates. Measures that reflect one or more years of delay at the beginning of autistic students’ academic endeavors predict study dropout in the second year: age and the missingness of average grades in secondary education. These predictors could be related to long-term issues with earlier educational fit and inclusion.

Previous analysis of the same data on autistic students’ background characteristics showed that they were older when they enrolled for the first time in higher education (19, 95% CI: 18–21; Bakker et al., 2019). Higher age may reflect the long-term difficulties with educational inclusion that autistic students experience before transitioning to university: issues with social relationships and bullying, lack of appropriate support, self-advocacy and transition planning, and problems with executive skills resulting in delays in high school (Anderson et al., 2017; Auger, 2013; Pinder-Amaker, 2014; Zeedyk et al., 2019). The missingness of the average grade in secondary education (9.3%) reflects dropout from regular education. Previous research on the same data shows that over 10% of autistic students had an irregular, longer learning path through entrance exams instead of a regular high school exam toward the university, in contrast to 2.6% of students with other conditions and 2.1% of students without conditions (Bakker et al., 2019). This deviation from the conventional learning path demonstrates autistic students’ persistence and determination to participate in higher education but does not guarantee their success.

### Prediction of third-year success

Prediction of third-year success concerns dropout rates, delays, and degree completion. The third group of predictors, related to autistic students’ earlier academic performance, also becomes important: the average grade in secondary education in general and in math. Again, the measured age reflects obstacles that have resulted in one or more years of delay at the beginning of autistic students’ academic careers. It predicts study dropout or delays in their bachelor’s program after 3 years. The missingness of the average grade in secondary education is less important, as students with irregular learning paths most likely have dropped out in the second year. Predictors for degree completion are the average grade in secondary education in general and in math, which indicate academic performance in secondary education. Average grades are better predictors of success than IQ since they capture aspects of personalities that have independent predictive power beyond IQ (Borghans et al., 2016). Furthermore, grade performance may be influenced by institutional preferences for particular styles of academic behavior (Tinto, 1975) or access to academic accommodations. Both grade predictors show that intellectual capabilities and institutional fit are equally crucial to autistic students’ academic success (Tinto, 1987, 2012; Van Rooij et al., 2018).

### Comparison of important predictors

Comparing prediction patterns of autistic students’ success to their peers’ success shows that, to a limited extent, these prediction patterns are unique to autistic students’ success. First-year success predictors are similar to their peers. However, a parallel program is most predictive for autistic students, which could imply that the personal capacity of autistic students and issues with transition are more important than study choice or academic performance-related predictors (Van den Broek et al., 2013).

Second-year success predictors of autistic students are more similar to students with other conditions success’ and appear to reflect long-term issues with an educational fit for both groups. In addition, predictors related to irregular learning paths reflect earlier education participation issues.

Third-year success predictors show that long-term issues with educational fit persist, resulting in delays similar to students with other conditions. On the other hand, and consistent with earlier findings, earlier academic performance, expressed as average grades in secondary education, is equally vital for autistic students’ long-term academic success as for their peers’ (Flegenheimer & Scherf, 2021; Gelbar et al., 2015; Gurbuz et al., 2019). As most autistic students in this study were enrolled in STEM programs (52.6%), the average grade in math (6.0, 95% CI: 6.0–7.0) is, compared to their peers, an additional predictor for degree completion. Autistic students’ talent for systemizing could explain their preponderance for STEM programs compared to students with other disabilities or neurotypical students (Wei et al., 2017). Regional influences are unlikely to play a part (Roelfsema et al., 2012).

### Limitations and future directions

Potential constraints of the present study must be acknowledged. PSW eliminates confounding by observed variables but can be biased if unmeasured factors predict outcomes that differ between autistic students and their peers. However, all causal modeling approaches that use observational student data have to deal with this constraint (McCaffrey et al., 2013). Therefore, this limitation is not specific to PSW.

Furthermore, we studied autistic students who applied for academic accommodations, which could bias results to more positive outcomes, as students who receive support are more likely to persist and complete a degree (Newman et al., 2018; Sarid et al., 2020). However, in this study, this applies to both autistic students and students with other conditions. Still, support should consider students who are either undiagnosed at the start of their university studies or who decide not to make their diagnosis known to the university, who may need additional attention.

Next, the kappa of all models for autistic students is fair (Landis & Koch, 1977). However, the optimal model on autistic students’ success after 3 years, with an accuracy of 64.3%, outperformed the NIR of the outcome measure by 21.4%. Including students’ results from early in their studies in predictive modeling is likely to improve accuracy. Since we wanted to use predictors available before the beginning of a student’s academic studies, we did not use these measures. More research into predictive models using results from early in autistic students’ studies is necessary.

We removed outliers, which implies that the prediction models do not cover extreme cases. Although we have found these models to be highly predictive of success, when supporting autistic students, one should also “expect the unexpected.” Additional factors, such as providing high-quality and appropriate support, the presence or absence of disability stigma during the student’s university studies, and social acceptance and support from academic peers (Anderson et al., 2017), could play a part. Field-specific factors that involve internships and research projects might also have influenced success (Büscher-Touwen et al., 2018). However, these features were not available. Additional modeling on possible differences between autistic students and students within different categories of disabilities, such as physical disabilities and mental health, would be of interest. However, the number of data points was too low. Further research into the influence of these aspects is needed.

Finally, although this is a large dataset, sample sizes are still relatively small, which lowers the predictive power of these models. To prevent bias as much as possible, we applied appropriate methods (Hellas et al., 2018; Vabalas et al., 2019). More research with larger samples is needed.

### Significance

To our knowledge, this is the first population study to use PSW in combination with predictive modeling to predict the academic success of autistic students compared to a major control group of students with other conditions and no conditions. This innovative methodological approach demonstrates that the academic success of autistic students, with the possible benefits of academic accommodations, can be predicted. Autistic students with irregular pre-educational study paths are more prone to study delays or dropping out. The risk of such events happening can be predicted based on information easily accessible in most institutions, including age and average math grades in secondary education. Predictive modeling can help talented students who might have a higher chance of failure to complete college. In addition, universities could further tailor transition and support programs (Nachman et al., 2022), such as summer transition programs (Hotez et al., 2018), and personal or peer transition coaching (Rando et al., 2016), to the specific students’ needs. These additional programs could also benefit many students with health conditions or non-traditional backgrounds.

### Compliance with ethical standards

All procedures performed in studies involving human participants were in accordance with the ethical standards of the institutional and/or national research committee and with the 1964 Helsinki Declaration and its later amendments or comparable ethical standards. For this type of study, formal consent is not required.

## Supplemental Material

sj-pdf-1-aut-10.1177_13623613221146439 – Supplemental material for Predicting academic success of autistic students in higher education Click here for additional data file. Supplemental material, sj-pdf-1-aut-10.1177_13623613221146439 for Predicting academic success of autistic students in higher education by Theo Bakker, Lydia Krabbendam, Sandjai Bhulai, Martijn Meeter and Sander Begeer in Autism

## Appendix 1

**Table 5.** Description of variables, measurement scales, and application.

Category	Variables	Measurement scales	Application
Conditions	Research group	Autism spectrum disorder (AS), Other	PSW, PM
		conditions (OC), No conditions (NC)	
Demographics	Gender	Female, Male	PSW, PM
	Age (in years)	Age	PSW, PM
Secondary education	Regular pre-education	TRUE = High school VWO, Vocational	PM
		foundation year, Degree in higher	
		education, or Foreign degree,	
		FALSE = Other pre-education	
Secondary education examination grades	Average grade secondary education	1–10	PM
	
	Average grade math secondary	1–10	PSW, PM
	education		
Enrollment	Cohort	2010, 2011, 2012, 2013, 2014, 2015,	PSW
		2016	
	Days between application and	336 to −336	PM
	September 1		
	Parallel program	TRUE = Yes, FALSE = No	PM
	STEM	TRUE = Yes, FALSE = No	PM
Student success	Success after 3 years	Degree, Dropout, Re-enrolled	PM

AS: autistic students; OC: students with other conditions; NC: students with no recorded conditions; PM: predictive modeling; PSW: propensity score weighting; VWO: Voorbereidend Wetenschappelijk Onderwijs.

## References

[bibr1-13623613221146439] Accardo A. L. Bean K. Cook B. Gillies A. Edgington R. Kuder S. J. Bomgardner E. M. (2019). College access, success and equity for students on the autism spectrum. Journal of Autism and Developmental Disorders, 49(12), 4877–4890. 10.1007/s10803-019-04205-8 31482372

[bibr2-13623613221146439] Alyahyan E. Düştegör D. (2020). Predicting academic success in higher education: Literature review and best practices. International Journal of Educational Technology in Higher Education, 17(1), 3. 10.1186/s41239-020-0177-7

[bibr3-13623613221146439] American Psychiatric Association. (2013). Diagnostic and statistical manual of mental disorders: DSM-5 (5th ed.).

[bibr4-13623613221146439] Anderson A. H. (2018). Perspectives of university students with autism spectrum disorder. Journal of Autism and Developmental Disorders, 48(3), 651–665. 10.1007/s10803-017-3257-3 28756552

[bibr5-13623613221146439] Anderson A. H. Stephenson J. Carter M. (2017). A systematic literature review of the experiences and supports of students with autism spectrum disorder in post-secondary education. Research in Autism Spectrum Disorders, 39, 33–53. 10.1016/j.rasd.2017.04.002

[bibr6-13623613221146439] Anderson A. H. Stephenson J. Carter M. Carlon S. (2019). A systematic literature review of empirical research on postsecondary students with autism spectrum disorder. Journal of Autism and Developmental Disorders, 49(4), 1531–1558. 10.1007/s10803-018-3840-2 30536219

[bibr7-13623613221146439] Auger R. (2013). Autism Spectrum Disorders: A research review for school counselors. Professional School Counseling, 16(4), 256–268. 10.1177/2156759X12016002S02

[bibr8-13623613221146439] Austin P. C. (2011). An introduction to propensity score methods for reducing the effects of confounding in observational studies. Multivariate Behavioral Research, 46(3), 399–424. 10.1080/00273171.2011.568786 21818162 PMC3144483

[bibr9-13623613221146439] Bakker T. Krabbendam L. Bhulai S. Begeer S. (2019). Background and Enrollment Characteristics of Students with Autism from Secondary Education to Higher Education. Research in Autism Spectrum Disorders, 67, 1–12. 10.1016/j.rasd.2019.101424

[bibr10-13623613221146439] Bakker T. Krabbendam L. Bhulai S. Begeer S. (2020). First-year progression and retention of students with autism in higher education: A propensity score weighted population study. Autism in Adulthood, 2(4), 307–316. 10.1089/aut.2019.0053

[bibr11-13623613221146439] Bakker T. Krabbendam L. Bhulai S. Meeter M. Begeer S. (2021). Predicting student success in students with autism in higher education: A longitudinal study. https://osf.io/hg6ry 10.1177/13623613221146439 PMC10374996 36602222

[bibr12-13623613221146439] Beardon L. Martin N. Woolsey I. (2009). What do students with Asperger syndrome or high-functioning autism want at college and university? (in their own words). Good Autism Practice, 10(2), 35–42.

[bibr13-13623613221146439] Biau G. Scornet E. (2016). A random forest guided tour. Test, 25(2), 197–227. 10.1007/s11749-016-0481-7

[bibr14-13623613221146439] Bonifro F. D. Gabbrielli M. Lisanti G. Zingaro S. P. (2020). Student dropout prediction. In Bittencourt I. Cukurova M. Muldner K. Luckin R. Millán E. (Eds.), Artificial intelligence in education. AIED 2020. Lecture notes in computer science (Vol. 12163, pp. 129–140). Springer. 10.1007/978-3-030-52237-7_11

[bibr15-13623613221146439] Borghans L. Golsteyn B. H. H. Heckman J. J. Humphries J. E. (2016). What grades and achievement tests measure. Proceedings of the National Academy of Sciences, 113(47), 13354–13359. 10.1073/pnas.1601135113 PMC5127298 27830648

[bibr16-13623613221146439] Breiman L. (1996). Bagging predictors. Machine Learning, 24(2), 123–140. 10.1007/bf00058655

[bibr17-13623613221146439] Büscher-Touwen M. Groot M. D. Hal L. V. (2018). Mind the gap between higher education and the labour market for students with a disability in the Netherlands: A research agenda. Social Inclusion, 6(4), 149–149. 10.17645/si.v6i4.1655

[bibr18-13623613221146439] Cage E. De Andres M. Mahoney P. (2020). Understanding the factors that affect university completion for autistic people. Research in Autism Spectrum Disorders, 72, 101519. 10.1016/j.rasd.2020.101519

[bibr19-13623613221146439] Centers for Disease Control and Prevention. (2022). Data & statistics on autism spectrum disorder. https://www.cdc.gov/ncbddd/autism/data.html

[bibr20-13623613221146439] Chiang H.-M. Cheung Y. K. Hickson L. Xiang R. Tsai L. Y. (2012). Predictive factors of participation in postsecondary education for high school leavers with autism. Journal of Autism and Developmental Disorders, 42(5), 685–696. 10.1007/s10803-011-1297-7 21618065

[bibr21-13623613221146439] Chown N. Baker-Rogers J. Hughes L. Cossburn K. Byrne P. (2016). The ‘High Achievers Project’: An assessment of the support for students with autism attending UK universities. Journal of Further and Higher Education, 42(6), 1–18. 10.1080/0309877X.2017.1323191

[bibr22-13623613221146439] Cox B. E. Edelstein J. Brogdon B. Roy A. (2021). Navigating challenges to facilitate success for college students with autism. Journal of Higher Education, 92, 1–27.

[bibr23-13623613221146439] European Commission. (n.d.). The Bologna process and the European higher education area. https://ec.europa.eu/education/policies/higher-education/bologna-process-and-european-higher-education-area%5C_en

[bibr24-13623613221146439] Flegenheimer C. Scherf K. S. (2021). College as a developmental context for emerging adulthood in autism: A systematic review of what we know and where we go from here. Journal of Autism and Developmental Disorders, 52, 2075–2097. 10.1007/s10803-021-05088-4 34060001 PMC8720487

[bibr25-13623613221146439] Francis G. Duke J. Chiu C. (2017). The college road trip: Supporting college success for students with autism. Division of Autism and Developmental Disabilities Online Journal, 1, 20–35.

[bibr26-13623613221146439] Friedman J. H. (2002). Stochastic gradient boosting. Computational Statistics & Data Analysis, 38(4), 367–378. 10.1016/s0167-9473(01)00065-2

[bibr27-13623613221146439] Gelbar N. W. Shefcyk A. Reichow B. (2015). A comprehensive survey of current and former college students with autism spectrum disorders. Yale Journal of Biology and Medicine, 88, 45–68. 25745374 PMC4345538

[bibr28-13623613221146439] Gelbar N. W. Smith I. Reichow B. (2014). Systematic review of articles describing experience and supports of individuals with autism enrolled in college and university programs. Journal of Autism and Developmental Disorders, 44(10), 2593–2601. 10.1007/s10803-014-2135-5 24816943

[bibr29-13623613221146439] Gillespie-Lynch K. Bublitz D. Donachie A. Wong V. Brooks P. J. D’Onofrio J. (2017). “For a long time our voices have been hushed”: Using student perspectives to develop supports for neurodiverse college students. Frontiers in Psychology, 8, 544. 10.3389/fpsyg.2017.00544 28458645 PMC5394111

[bibr30-13623613221146439] Greene W. H. (2012). Econometric analysis (7th ed.). Pearson Education.

[bibr31-13623613221146439] Greenwell B. Boehmke B. Jay C. , & GBM Developers. (2020). Gbm: Generalized boosted regression models [R package version 2.1.8]. https://CRAN.R-project.org/package=gbm

[bibr32-13623613221146439] Greifer N. (2019). Cobalt: Covariate balance tables and plots [R package version 3.6.1]. https://CRAN.R-project.org/package=cobalt

[bibr33-13623613221146439] Gurbuz E. Hanley M. Riby D. M. (2019). University students with autism: The social and academic experiences of university in the UK. Journal of Autism and Developmental Disorders, 49(2), 617–631. 10.1007/s10803-018-3741-4 30173311 PMC6373295

[bibr34-13623613221146439] Hellas A. Ihantola P. Petersen A. Ajanovski V. V. Gutica M. Hynninen T. Knutas A. Leinonen J. Messom C. Liao S. N. (2018). Predicting academic performance: A systematic literature review. In Proceedings companion of the 23rd annual ACM conference on innovation and technology in computer science education (pp. 175–199). 10.1145/3293881.3295783

[bibr35-13623613221146439] Hoekstra R. (2018). Prevalentie. In Geurts H. Sizoo B. Noens I. (Eds.), Autismespectrumstoornis (pp. 19-27). Bohn Stafleu van Loghum. 10.1007/978-90-368-2042-4_2

[bibr36-13623613221146439] Hotez E. Shane-Simpson C. Obeid R. DeNigris D. Siller M. Costikas C. Gillespie-Lynch K. (2018). Designing a summer transition program for incoming and current college students on the autism spectrum: A participatory approach. Frontiers in Psychology, 9, 46. 10.3389/fpsyg.2018.00046 29487547 PMC5816926

[bibr37-13623613221146439] Hyde K. K. Novack M. N. LaHaye N. Parlett-Pelleriti C. Anden R. Dixon D. R. Linstead E. (2019). Applications of supervised machine learning in autism spectrum disorder research: A review. Review Journal of Autism and Developmental Disorders, 6(2), 128–146. 10.1007/s40489-019-00158-x

[bibr38-13623613221146439] Jansen D. Petry K. Ceulemans E. Noens I. Baeyens D. (2016). Functioning and participation problems of students with ASD in higher education: Which reasonable accommodations are effective? European Journal of Special Needs Education, 32(1), 71–88. 10.1080/08856257.2016.1254962

[bibr39-13623613221146439] Jia P. Maloney T. (2015). Using predictive modelling to identify students at risk of poor university outcomes. Higher Education, 70(1), 127–149. 10.1007/s10734-014-9829-7

[bibr40-13623613221146439] Khan A. Ghosh S. K. (2021). Student performance analysis and prediction in classroom learning: A review of educational data mining studies. Education and Information Technologies, 26(1), 205–240. 10.1007/s10639-020-10230-3

[bibr41-13623613221146439] Kuhn M. (2015). A short introduction to the caret package. R Foundation for Statistical Computing, 1, 1–10.

[bibr42-13623613221146439] Kuhn M. (2021). Caret: Classification and regression training [R package version 6.0-88]. https://CRAN.R-project.org/package=caret

[bibr43-13623613221146439] Kuhn M. Johnson K. (2013). Applied predictive modeling. Springer. 10.1007/978-1-4614-6849-3

[bibr44-13623613221146439] Lambe S. Russell A. Butler C. Fletcher S. Ashwin C. Brosnan M. (2018). Autism and the transition to university from the student perspective. Autism, 10(2), 1362361318803935. 10.1177/1362361318803935 30582345

[bibr45-13623613221146439] Landis J. R. Koch G. G. (1977). The measurement of observer agreement for categorical data. Biometrics, 33(1), 159–174. 10.2307/2529310 843571

[bibr46-13623613221146439] Leppink J. (2020). The art of modelling the learning process, uniting educational research and practice. Springer. 10.1007/978-3-030-43082-5

[bibr47-13623613221146439] Loh W.-Y. (2011). Classification and regression trees. Wiley Interdisciplinary Reviews: Data Mining and Knowledge Discovery, 1(1), 14–23. 10.1002/widm.8 PMC3329156 22523608

[bibr48-13623613221146439] McCaffrey D. F. Griffin B. A. Almirall D. Slaughter M. E. Ramchand R. Burgette L. F. (2013). A tutorial on propensity score estimation for multiple treatments using generalized boosted models. Statistics in Medicine, 32(19), 3388–3414. 10.1002/sim.5753 23508673 PMC3710547

[bibr49-13623613221146439] Madaus J. W. Gelbar N. Dukes I. I. I. L. L. Taconet A. Faggella-Luby M. (2020). Are there predictors of success for students with disabilities pursuing postsecondary education? Career Development and Transition for Exceptional Individuals, 44(4), 191–202. 10.1177/2165143420976526

[bibr50-13623613221146439] Meinel C. Pérez-Sanagustín M. Specht M. Ogan A. Yu R. Lee H. Kizilcec R. F. (2021). Should college dropout prediction models include protected attributes? In Proceedings of the eighth ACM conference on learning @ Scale (pp. 91–100). 10.1145/3430895.3460139

[bibr51-13623613221146439] Morgan C. D. (2018). The academic performance of college students with autism spectrum disorder [Doctoral dissertation]. Fairleigh Dickinson University.

[bibr52-13623613221146439] Nachman B. R. McDermott C. T. Cox B. E. (2022). Brief Report: Autism-specific college support programs: Differences across geography and institutional type. Journal of Autism and Developmental Disorders, 52(2), 863–870. 10.1007/s10803-021-04958-1 33770324 PMC8813697

[bibr53-13623613221146439] Namoun A. Alshanqiti A. (2020). Predicting student performance using data mining and learning analytics techniques: A systematic literature review. Applied Sciences, 11(1), 237. 10.3390/app11010237

[bibr54-13623613221146439] Newman L. A. Madaus J. W. Lalor A. R. Javitz H. S. (2018). Support receipt: Effect on postsecondary success of students with learning disabilities. Career Development and Transition for Exceptional Individuals, 42(1), 6–16. 10.1177/2165143418811288

[bibr55-13623613221146439] Nuske A. Rillotta F. Bellon M. Richdale A. (2019). Transition to higher education for students with autism: A systematic literature review. Journal of Diversity in Higher Education, 12(3), 280–295. 10.1037/dhe0000108

[bibr56-13623613221146439] Nuske H. J. McGhee Hassrick E. Bronstein B. Hauptman L. Aponte C. Levato L. Smith T. (2019). Broken bridges — new school transitions for students with autism spectrum disorder: A systematic review on difficulties and strategies for success. Autism, 23(2), 306–325. 10.1177/1362361318754529 29458258

[bibr57-13623613221146439] Peters A. Hothorn T. (2021). Ipred: Improved predictors [R package version 0.9-11]. https://CRAN.R-project.org/package=ipred

[bibr58-13623613221146439] Peterson R. A. Cavanaugh J. E. (2019). Ordered quantile normalization: A semiparametric transformation built for the cross-validation era. Journal of Applied Statistics, 47, 2312–2327. 10.1080/02664763.2019.1630372 35707424 PMC9042069

[bibr59-13623613221146439] Pinder-Amaker S. (2014). Identifying the unmet needs of college students on the autism spectrum. Harvard Review of Psychiatry, 22(2), 125–137. 10.1097/HRP.0000000000000032 24614767

[bibr60-13623613221146439] Pingry O’Neill L. N. Markward M. J. French J. P . (2012). Predictors of graduation among college students with disabilities. Journal of Postsecondary Education and Disability, 25(1), 21–36.

[bibr61-13623613221146439] Rahman M. M. Usman O. L. Muniyandi R. C. Sahran S. Mohamed S. Razak R. A. (2020). A review of machine learning methods of feature selection and classification for autism spectrum disorder. Brain Sciences, 10(12), 949. 10.3390/brainsci10120949 33297436 PMC7762227

[bibr62-13623613221146439] Rando H. Huber M. Oswald G. (2016). An academic coaching model intervention for college students on the autism spectrum. Journal of Postsecondary Education and Disability, 3(29), 257–262.

[bibr63-13623613221146439] R Core Team. (2017). R: A language and environment for statistical computing. R Foundation for Statistical Computing. https://www.R-project.org/

[bibr64-13623613221146439] Robertson S. M. Ne’eman A. D. (2008). Autistic acceptance, the college campus, and technology: Growth of neurodiversity in society and academia. Disability Studies Quarterly, 28(4), 146. 10.18061/dsq.v28i4.146

[bibr65-13623613221146439] Roelfsema M. T. Hoekstra R. A. Allison C. Wheelwright S. Brayne C. Matthews F. E. Baron-Cohen S. (2012). Are autism spectrum conditions more prevalent in an information-technology region? A school-based study of three regions in the Netherlands. Journal of Autism and Developmental Disorders, 42(5), 734–739. 10.1007/s10803-011-1302-1 21681590

[bibr66-13623613221146439] Sarid M. Meltzer Y. Raveh M. (2020). Academic achievements of college graduates with learning disabilities vis-a-vis admission criteria and academic support. Journal of Learning Disabilities, 53(1), 60–74. 10.1177/0022219419884064 31674261

[bibr67-13623613221146439] Shmueli G. (2010). To explain or to predict? Statistical Science, 25(3), 289–310. 10.1214/10-sts330

[bibr68-13623613221146439] Shmueli G. Koppius O. R. (2011). Predictive analytics in information systems research. MIS Quarterly, 35(3), 553. 10.2307/23042796

[bibr69-13623613221146439] Shmulsky S. Gobbo K. Donahue A. T. Banerjee M. (2017). College students who have ASD: Factors related to first year performance. Journal of Postsecondary Education and Disability, 30(4), 375–384

[bibr70-13623613221146439] STEM Designated Degree Program List. (2016). www.ice.gov/sites/default/files/documents/Document/2016/stem-list.pdf

[bibr71-13623613221146439] Thabtah F. (2018). Machine learning in autistic spectrum disorder behavioral research: A review and ways forward. Informatics for Health and Social Care, 44(3), 1–20. 10.1080/17538157.2017.1399132 29436887

[bibr72-13623613221146439] Therneau T. Atkinson B. (2019). Rpart: Recursive partitioning and regression trees [R package version 4.1-15]. https://CRAN.R-project.org/package=rpart

[bibr73-13623613221146439] Tinto V. (1975). Dropout from higher education: A theoretical synthesis of recent research. Review of Educational Research, 45(1), 89–125.

[bibr74-13623613221146439] Tinto V. (1987). Leaving college: Rethinking the causes and cures of student attrition. University of Chicago Press.

[bibr75-13623613221146439] Tinto V. (2012). Completing college: Rethinking institutional action. University of Chicago Press.

[bibr76-13623613221146439] Vabalas A. Gowen E. Poliako E. Casson A. J. (2019). Machine learning algorithm validation with a limited sample size. PLOS ONE, 14(11), e0224365. 10.1371/journal.pone.0224365 PMC6837442 31697686

[bibr77-13623613221146439] Van den Broek A. Muskens M. Winkels J . (2013). Studeren met een functiebeperking 2012 (tech. rep.). ResearchNed/ITS Nijmegen.

[bibr78-13623613221146439] Van den Broek A. Wartenbergh F. Bendig-Jacobs J. Tholen R. Duysak S. Nooij J . (2017). Monitor Beleidsmaatregelen 2016-2017 (tech. rep.). ResearchNed/ITS Nijmegen. https://www.scienceguide.nl/media/1937056/monitor%5C_beleidsmaatregelen%5C_2016-2017.pdf

[bibr79-13623613221146439] Van Hees V. Moyson T. Roeyers H . (2015). Higher education experiences of students with autism spectrum disorder: Challenges, benefits and support needs. Journal of Autism and Developmental Disorders, 45(6), 1673–1688. 10.1007/s10803-014-2324-2 25448918

[bibr80-13623613221146439] Van Rooij E. Brouwer J. Fokkens-Bruinsma M. Jansen E. Donche V. Noyens D . (2018). A systematic review of factors related to first-year students’ success in Dutch and Flemish higher education. Pedagogische Studiën, 94(5), 360–405

[bibr81-13623613221146439] Venables W. N. Ripley B. D. (2002). Modern applied statistics with S (4th ed.). Springer. https://www.stats.ox.ac.uk/pub/MASS4/

[bibr82-13623613221146439] Vincent J. (2019). It’s the fear of the unknown: Transition from higher education for young autistic adults. Autism, 10(7), 1362361318822498. 10.1177/1362361318822498 30632780

[bibr83-13623613221146439] Ward D. Webster A. (2018). Understanding the lived experiences of university students with autism spectrum disorder (ASD): A phenomenological study. International Journal of Disability, Development and Education, 65, 373–392. 10.1080/1034912x.2017.1403573

[bibr84-13623613221146439] Wei X. Yu J. W. Shattuck P. Blackorby J. (2017). High school math and science preparation and postsecondary STEM participation for students with an autism spectrum disorder. Focus on Autism and Other Developmental Disabilities, 32(2), 83–92. 10.1177/1088357615588489

[bibr85-13623613221146439] White S. W. Elias R. Capriola-Hall N. N. Smith I. C. Conner C. M. Asselin S. B. Howlin P. Getzel E. E. Mazefsky C. A. (2017). Development of a college transition and support program for students with autism spectrum disorder. Journal of Autism and Developmental Disorders, 13(1), 1–7. 10.1007/s10803-017-3236-8 PMC5922249 28685409

[bibr86-13623613221146439] Wright M. N. Ziegler A. (2017). ranger: A fast implementation of random forests for high dimensional data in C++ and R. Journal of Statistical Software, 77(1), 1–17. 10.18637/jss.v077.i01

[bibr87-13623613221146439] Zeedyk S. M. Bolourian Y. Blacher J. (2019). University life with ASD: Faculty knowledge and student needs. Autism, 23(3), 726–736. 10.1177/1362361318774148 29788749

[bibr88-13623613221146439] Zeedyk S. M. Tipton L. A. Blacher J. (2016). Educational supports for high functioning youth with ASD: The postsecondary pathway to college. Focus on Autism and Other Developmental Disabilities, 31(1), 37–48. 10.1177/1088357614525435

