# Supplementary material for: Predicting academic success of autistic students in higher education
Source: Autism. 2023 Jan 5;27(6):1803–16. doi: 10.1177/13623613221146439 (PMC10374996; doi:10.1177/13623613221146439)
Supplement: sj-pdf-1-aut-10.1177_13623613221146439 – Supplemental material for Predicting academic success of autistic students in higher education [file sj-pdf-1-aut-10.1177_13623613221146439.pdf]

Supplementary Material

Table S1

Model performance for the three participant groups

Outcome	Group	Model	Split	Set	Accuracy	κ	NIR	p.value
Dropout after 1 year	AS	CART	60/60%	Test	76.3% (59.8%-88.6%)	0.00	76.3%	0.588
				Train	76.3% (63.4%-86.4%)	0.00	76.3%	0.571
			70/30%	Test	78.6% (59.0%-91.7%)	0.00	78.6%	0.607
				Train	75.4% (63.5%-84.9%)	0.00	75.4%	0.565
			80/20%	Test	77.8% (52.4%-93.6%)	0.00	77.8%	0.630
				Train	75.9% (65.0%-84.9%)	0.00	75.9%	0.561
Dropout after 1 year	AS	RF	60/60%	Test	76.3% (59.8%-88.6%)	0.00	76.3%	0.588
				Train	79.7% (67.2%-89.0%)	0.20	76.3%	0.331
			70/30%	Test	78.6% (59.0%-91.7%)	0.00	78.6%	0.607
				Train	82.6% (71.6%-90.7%)	0.39	75.4%	0.101
			80/20%	Test	77.8% (52.4%-93.6%)	0.00	77.8%	0.630
				Train	79.7% (69.2%-88.0%)	0.22	75.9%	0.260
Dropout after 1 year	AS	PMR	60/60%	Test	73.7% (56.9%-86.6%)	-0.05	76.3%	0.724
				Train	83.1% (71.0%-91.6%)	0.38	76.3%	0.141
			70/30%	Test	71.4% (51.3%-86.8%)	0.15	78.6%	0.873
				Train	79.7% (68.3%-88.4%)	0.28	75.4%	0.246
			80/20%	Test	83.3% (58.6%-96.4%)	0.34	77.8%	0.409
				Train	77.2% (66.4%-85.9%)	0.12	75.9%	0.457
Dropout after 1 year	AS	SGB	60/60%	Test	73.7% (56.9%-86.6%)	-0.05	76.3%	0.724
				Train	81.4% (69.1%-90.3%)	0.29	76.3%	0.226
			70/30%	Test	75.0% (55.1%-89.3%)	-0.07	78.6%	0.762
				Train	78.3% (66.7%-87.3%)	0.21	75.4%	0.345
			80/20%	Test	66.7% (41.0%-86.7%)	-0.17	77.8%	0.916
				Train	77.2% (66.4%-85.9%)	0.08	75.9%	0.457
Dropout after 1 year	AS	BCART	60/60%	Test	73.7% (56.9%-86.6%)	0.14	76.3%	0.724
				Train	98.3% (90.9%-100.0%)	0.95	76.3%	< 0.001 ***
			70/30%	Test	82.1% (63.1%-93.9%)	0.24	78.6%	0.426
				Train	100.0% (94.8%-100.0%)	1.00	75.4%	< 0.001 ***
			80/20%	Test	66.7% (41.0%-86.7%)	-0.17	77.8%	0.916
				Train	98.7% (93.1%-100.0%)	0.96	75.9%	< 0.001 ***
Dropout after 1 year	OC	CART	60/60%	Test	77.6% (74.9%-80.2%)	0.07	79.5%	0.930
				Train	81.0% (78.8%-83.0%)	0.19	79.4%	0.079 .
			70/30%	Test	79.5% (76.4%-82.4%)	0.00	79.5%	0.522
				Train	79.4% (77.4%-81.4%)	0.00	79.4%	0.514
			80/20%	Test	78.2% (74.3%-81.9%)	-0.01	79.5%	0.771
				Train	80.3% (78.4%-82.1%)	0.10	79.5%	0.191
Dropout after 1 year	OC	RF	60/60%	Test	79.5% (76.8%-82.0%)	0.00	79.5%	0.519
				Train	79.4% (77.2%-81.5%)	0.00	79.4%	0.516

continued on next page

Table S1 – *continued from previous page*

Outcome	Group	Model	Split	Set	Accuracy	κ	NIR	p.value
			70/30%	Test	79.5% (76.4%-82.4%)	0.00	79.5%	0.522
				Train	79.4% (77.4%-81.4%)	0.00	79.4%	0.514
			80/20%	Test	79.5% (75.6%-83.0%)	0.00	79.5%	0.527
				Train	79.5% (77.6%-81.2%)	0.00	79.5%	0.513
Dropout after 1 year	OC	PMR	60/60%	Test	79.5% (76.8%-82.0%)	0.00	79.5%	0.519
				Train	79.4% (77.2%-81.5%)	0.00	79.4%	0.516
			70/30%	Test	79.5% (76.4%-82.4%)	0.00	79.5%	0.522
				Train	79.4% (77.4%-81.4%)	0.00	79.4%	0.514
			80/20%	Test	79.5% (75.6%-83.0%)	0.00	79.5%	0.527
				Train	79.5% (77.6%-81.2%)	0.00	79.5%	0.513
Dropout after 1 year	OC	SGB	60/60%	Test	79.7% (77.0%-82.2%)	0.02	79.5%	0.455
				Train	79.5% (77.3%-81.6%)	0.01	79.4%	0.490
			70/30%	Test	79.0% (75.8%-81.9%)	-0.00	79.5%	0.664
				Train	79.7% (77.7%-81.6%)	0.03	79.4%	0.418
			80/20%	Test	79.5% (75.6%-83.0%)	0.00	79.5%	0.527
				Train	79.9% (78.0%-81.6%)	0.04	79.5%	0.337
Dropout after 1 year	OC	BCART	60/60%	Test	74.9% (72.0%-77.6%)	0.05	79.5%	1.000
				Train	99.7% (99.2%-99.9%)	0.99	79.4%	< 0.001 ***
			70/30%	Test	73.4% (70.0%-76.6%)	-0.01	79.5%	1.000
				Train	99.6% (99.2%-99.9%)	0.99	79.4%	< 0.001 ***
			80/20%	Test	75.3% (71.2%-79.1%)	0.04	79.5%	0.989
				Train	99.5% (99.0%-99.7%)	0.98	79.5%	< 0.001 ***
Dropout after 1 year	NC	CART	60/60%	Test	72.8% (71.9%-73.7%)	0.09	73.0%	0.648
				Train	74.2% (73.5%-74.9%)	0.14	73.0%	< 0.001 ***
			70/30%	Test	73.0% (72.0%-74.0%)	0.08	73.0%	0.496
				Train	74.2% (73.6%-74.9%)	0.12	73.0%	< 0.001 ***
			80/20%	Test	72.6% (71.4%-73.9%)	0.06	73.0%	0.715
				Train	73.8% (73.2%-74.4%)	0.10	73.0%	0.005 **
Dropout after 1 year	NC	RF	60/60%	Test	72.8% (71.9%-73.7%)	0.04	73.0%	0.665
				Train	76.5% (75.8%-77.2%)	0.18	73.0%	< 0.001 ***
			70/30%	Test	73.0% (72.0%-74.0%)	0.00	73.0%	0.496
				Train	73.0% (72.4%-73.7%)	0.00	73.0%	0.428
			80/20%	Test	72.6% (71.3%-73.9%)	0.02	73.0%	0.737
				Train	75.7% (75.1%-76.4%)	0.15	73.0%	< 0.001 ***
Dropout after 1 year	NC	PMR	60/60%	Test	73.0% (72.1%-73.8%)	0.00	73.0%	0.533
				Train	73.0% (72.3%-73.7%)	0.01	73.0%	0.467
			70/30%	Test	73.0% (72.0%-74.0%)	0.00	73.0%	0.496
				Train	73.0% (72.3%-73.7%)	0.00	73.0%	0.476
			80/20%	Test	72.8% (71.5%-74.1%)	-0.00	73.0%	0.622
				Train	73.1% (72.4%-73.7%)	0.01	73.0%	0.389
Dropout after 1 year	NC	SGB	60/60%	Test	72.7% (71.8%-73.6%)	0.03	73.0%	0.728
				Train	73.2% (72.4%-73.9%)	0.05	73.0%	0.331

continued on next page

Table S1 – *continued from previous page*

Outcome	Group	Model	Split	Set	Accuracy	κ	NIR	p.value
			70/30%	Test	73.2% (72.1%-74.2%)	0.02	73.0%	0.381
				Train	73.0% (72.3%-73.6%)	0.01	73.0%	0.518
			80/20%	Test	72.9% (71.6%-74.2%)	0.02	73.0%	0.559
				Train	73.0% (72.4%-73.7%)	0.02	73.0%	0.439
Dropout after 1 year	NC	BCART	60/60%	Test	68.0% (67.1%-68.9%)	0.09	73.0%	1.000
				Train	98.1% (97.9%-98.3%)	0.95	73.0%	< 0.001 ***
			70/30%	Test	67.3% (66.2%-68.4%)	0.09	73.0%	1.000
				Train	98.2% (98.0%-98.4%)	0.95	73.0%	< 0.001 ***
			80/20%	Test	66.6% (65.3%-68.0%)	0.07	73.0%	1.000
				Train	98.0% (97.8%-98.2%)	0.95	73.0%	< 0.001 ***
Dropout after 2 years	AS	CART	60/60%	Test	50.0% (33.4%-66.6%)	-0.12	63.2%	0.966
				Train	72.9% (59.7%-83.6%)	0.39	62.7%	0.067 .
			70/30%	Test	67.9% (47.6%-84.1%)	0.17	64.3%	0.429
				Train	73.9% (61.9%-83.7%)	0.37	62.3%	0.029 *
			80/20%	Test	63.2% (38.4%-83.7%)	0.00	63.2%	0.601
				Train	62.8% (51.1%-73.5%)	0.00	62.8%	0.551
Dropout after 2 years	AS	RF	60/60%	Test	73.7% (56.9%-86.6%)	0.36	63.2%	0.118
				Train	72.9% (59.7%-83.6%)	0.32	62.7%	0.067 .
			70/30%	Test	75.0% (55.1%-89.3%)	0.39	64.3%	0.162
				Train	73.9% (61.9%-83.7%)	0.36	62.3%	0.029 *
			80/20%	Test	63.2% (38.4%-83.7%)	0.13	63.2%	0.601
				Train	83.3% (73.2%-90.8%)	0.61	62.8%	< 0.001 ***
Dropout after 2 years	AS	PMR	60/60%	Test	73.7% (56.9%-86.6%)	0.38	63.2%	0.118
				Train	72.9% (59.7%-83.6%)	0.32	62.7%	0.067 .
			70/30%	Test	53.6% (33.9%-72.5%)	0.05	64.3%	0.914
				Train	76.8% (65.1%-86.1%)	0.47	62.3%	0.008 **
			80/20%	Test	63.2% (38.4%-83.7%)	0.13	63.2%	0.601
				Train	76.9% (66.0%-85.7%)	0.43	62.8%	0.006 **
Dropout after 2 years	AS	SGB	60/60%	Test	57.9% (40.8%-73.7%)	0.01	63.2%	0.801
				Train	76.3% (63.4%-86.4%)	0.44	62.7%	0.019 *
			70/30%	Test	64.3% (44.1%-81.4%)	0.15	64.3%	0.585
				Train	76.8% (65.1%-86.1%)	0.48	62.3%	0.008 **
			80/20%	Test	68.4% (43.4%-87.4%)	0.23	63.2%	0.414
				Train	70.5% (59.1%-80.3%)	0.27	62.8%	0.097 .
Dropout after 2 years	AS	BCART	60/60%	Test	57.9% (40.8%-73.7%)	0.04	63.2%	0.801
				Train	100.0% (93.9%-100.0%)	1.00	62.7%	< 0.001 ***
			70/30%	Test	50.0% (30.6%-69.4%)	-0.14	64.3%	0.960
				Train	100.0% (94.8%-100.0%)	1.00	62.3%	< 0.001 ***
			80/20%	Test	63.2% (38.4%-83.7%)	0.13	63.2%	0.601
				Train	100.0% (95.4%-100.0%)	1.00	62.8%	< 0.001 ***
Dropout after 2 years	OC	CART	60/60%	Test	73.0% (70.1%-75.8%)	0.01	74.4%	0.841

continued on next page

Table S1 – *continued from previous page*

Outcome	Group	Model	Split	Set	Accuracy	κ	NIR	p.value
				Train	75.4% (73.1%-77.6%)	0.09	74.4%	0.191
			70/30%	Test	73.3% (69.9%-76.5%)	0.01	74.4%	0.767
				Train	75.4% (73.3%-77.4%)	0.10	74.4%	0.178
			80/20%	Test	72.8% (68.6%-76.7%)	0.05	74.5%	0.814
				Train	76.4% (74.4%-78.3%)	0.17	74.3%	0.021 *
Dropout after 2 years	OC	RF	60/60%	Test	74.3% (71.4%-77.0%)	-0.00	74.4%	0.547
				Train	74.6% (72.2%-76.8%)	0.01	74.4%	0.442
			70/30%	Test	74.4% (71.0%-77.5%)	0.00	74.4%	0.520
				Train	74.4% (72.2%-76.4%)	0.00	74.4%	0.513
			80/20%	Test	74.5% (70.3%-78.3%)	0.00	74.5%	0.524
				Train	74.3% (72.3%-76.3%)	0.00	74.3%	0.512
Dropout after 2 years	OC	PMR	60/60%	Test	74.4% (71.5%-77.1%)	0.00	74.4%	0.517
				Train	74.4% (72.0%-76.6%)	0.00	74.4%	0.514
			70/30%	Test	74.4% (71.0%-77.5%)	0.00	74.4%	0.520
				Train	74.4% (72.2%-76.4%)	0.00	74.4%	0.513
			80/20%	Test	74.7% (70.5%-78.5%)	0.04	74.5%	0.482
				Train	74.8% (72.8%-76.7%)	0.04	74.3%	0.349
Dropout after 2 years	OC	SGB	60/60%	Test	74.0% (71.1%-76.7%)	0.07	74.4%	0.633
				Train	75.5% (73.2%-77.7%)	0.11	74.4%	0.160
			70/30%	Test	74.2% (70.9%-77.4%)	0.03	74.4%	0.554
				Train	74.7% (72.6%-76.8%)	0.05	74.4%	0.381
			80/20%	Test	74.1% (69.9%-77.9%)	0.01	74.5%	0.606
				Train	75.3% (73.3%-77.3%)	0.06	74.3%	0.167
Dropout after 2 years	OC	BCART	60/60%	Test	67.4% (64.3%-70.4%)	0.04	74.4%	1.000
				Train	99.9% (99.5%-100.0%)	1.00	74.4%	< 0.001 ***
			70/30%	Test	69.6% (66.1%-73.0%)	0.01	74.4%	0.998
				Train	99.6% (99.2%-99.9%)	0.99	74.4%	< 0.001 ***
			80/20%	Test	68.2% (63.8%-72.4%)	0.02	74.5%	0.999
				Train	99.6% (99.2%-99.8%)	0.99	74.3%	< 0.001 ***
Dropout after 2 years	NC	CART	60/60%	Test	68.9% (68.0%-69.8%)	0.13	68.5%	0.219
				Train	70.0% (69.3%-70.8%)	0.16	68.5%	< 0.001 ***
			70/30%	Test	69.3% (68.2%-70.3%)	0.12	68.5%	0.084 .
				Train	69.4% (68.7%-70.1%)	0.11	68.5%	0.007 **
			80/20%	Test	68.8% (67.4%-70.1%)	0.13	68.5%	0.362
				Train	70.1% (69.4%-70.7%)	0.17	68.5%	< 0.001 ***
Dropout after 2 years	NC	RF	60/60%	Test	69.3% (68.4%-70.2%)	0.11	68.5%	0.051 .
				Train	74.8% (74.1%-75.6%)	0.28	68.5%	< 0.001 ***
			70/30%	Test	69.8% (68.7%-70.9%)	0.12	68.5%	0.010 **
				Train	74.7% (74.1%-75.4%)	0.27	68.5%	< 0.001 ***
			80/20%	Test	69.6% (68.3%-70.9%)	0.12	68.5%	0.059 .
				Train	74.6% (74.0%-75.2%)	0.27	68.5%	< 0.001 ***
Dropout after 2 years	NC	PMR	60/60%	Test	69.1% (68.1%-70.0%)	0.14	68.5%	0.130

continued on next page

Table S1 – *continued from previous page*

Outcome	Group	Model	Split	Set	Accuracy	κ	NIR	p.value
				Train	69.5% (68.8%-70.3%)	0.15	68.5%	0.005 **
			70/30%	Test	69.8% (68.7%-70.9%)	0.15	68.5%	0.009 **
				Train	69.3% (68.6%-70.0%)	0.14	68.5%	0.011 *
			80/20%	Test	69.4% (68.1%-70.7%)	0.14	68.5%	0.089 .
				Train	69.5% (68.9%-70.2%)	0.15	68.5%	0.001 ***
Dropout after 2 years	NC	SGB	60/60%	Test	69.4% (68.5%-70.4%)	0.14	68.5%	0.026 *
				Train	70.0% (69.3%-70.8%)	0.15	68.5%	< 0.001 ***
			70/30%	Test	69.9% (68.9%-71.0%)	0.15	68.5%	0.005 **
				Train	70.1% (69.4%-70.8%)	0.15	68.5%	< 0.001 ***
			80/20%	Test	69.8% (68.5%-71.1%)	0.14	68.5%	0.030 *
				Train	70.4% (69.7%-71.0%)	0.17	68.5%	< 0.001 ***
Dropout after 2 years	NC	BCART	60/60%	Test	65.2% (64.2%-66.1%)	0.13	68.5%	1.000
				Train	98.9% (98.8%-99.1%)	0.97	68.5%	< 0.001 ***
			70/30%	Test	65.8% (64.7%-66.8%)	0.15	68.5%	1.000
				Train	98.7% (98.5%-98.9%)	0.97	68.5%	< 0.001 ***
			80/20%	Test	65.5% (64.1%-66.8%)	0.14	68.5%	1.000
				Train	98.7% (98.5%-98.8%)	0.97	68.5%	< 0.001 ***
Success after 3 years	AS	CART	60/60%	Test	39.5% (24.0%-56.6%)	0.05	42.1%	0.686
				Train	61.0% (47.4%-73.5%)	0.40	42.4%	0.003 **
			70/30%	Test	42.9% (24.5%-62.8%)	0.00	42.9%	0.572
				Train	42.0% (30.2%-54.5%)	0.00	42.0%	0.546
			80/20%	Test	42.1% (20.3%-66.5%)	0.00	42.1%	0.587
				Train	42.3% (31.2%-54.0%)	0.00	42.3%	0.543
Success after 3 years	AS	RF	60/60%	Test	50.0% (33.4%-66.6%)	0.15	42.1%	0.205
				Train	66.1% (52.6%-77.9%)	0.44	42.4%	< 0.001 ***
			70/30%	Test	64.3% (44.1%-81.4%)	0.40	42.9%	0.018 *
				Train	68.1% (55.8%-78.8%)	0.47	42.0%	< 0.001 ***
			80/20%	Test	63.2% (38.4%-83.7%)	0.38	42.1%	0.053 .
				Train	67.9% (56.4%-78.1%)	0.47	42.3%	< 0.001 ***
Success after 3 years	AS	PMR	60/60%	Test	36.8% (21.8%-54.0%)	0.04	42.1%	0.793
				Train	69.5% (56.1%-80.8%)	0.52	42.4%	< 0.001 ***
			70/30%	Test	57.1% (37.2%-75.5%)	0.27	42.9%	0.091 .
				Train	59.4% (46.9%-71.1%)	0.32	42.0%	0.003 **
			80/20%	Test	52.6% (28.9%-75.6%)	0.20	42.1%	0.241
				Train	59.0% (47.3%-70.0%)	0.31	42.3%	0.002 **
Success after 3 years	AS	SGB	60/60%	Test	42.1% (26.3%-59.2%)	0.00	42.1%	0.562
				Train	42.4% (29.6%-55.9%)	0.01	42.4%	0.550
			70/30%	Test	46.4% (27.5%-66.1%)	0.07	42.9%	0.421
				Train	44.9% (32.9%-57.4%)	0.05	42.0%	0.355
			80/20%	Test	47.4% (24.4%-71.1%)	0.10	42.1%	0.404
				Train	42.3% (31.2%-54.0%)	0.00	42.3%	0.543

continued on next page

Table S1 – *continued from previous page*

Outcome	Group	Model	Split	Set	Accuracy	κ	NIR	p.value
Success after 3 years	AS	BCART	60/60%	Test	34.2% (19.6%-51.4%)	-0.04	42.1%	0.876
				Train	100.0% (93.9%-100.0%)	1.00	42.4%	< 0.001 ***
			70/30%	Test	28.6% (13.2%-48.7%)	-0.15	42.9%	0.960
				Train	100.0% (94.8%-100.0%)	1.00	42.0%	< 0.001 ***
			80/20%	Test	15.8% (3.4%-39.6%)	-0.33	42.1%	0.997
				Train	100.0% (95.4%-100.0%)	1.00	42.3%	< 0.001 ***
Success after 3 years	OC	CART	60/60%	Test	45.7% (42.5%-48.9%)	0.06	48.1%	0.936
				Train	53.4% (50.8%-56.0%)	0.19	47.9%	< 0.001 ***
			70/30%	Test	48.1% (44.3%-51.8%)	0.00	48.1%	0.515
				Train	48.0% (45.6%-50.4%)	0.00	48.0%	0.510
			80/20%	Test	47.9% (43.3%-52.5%)	0.09	48.1%	0.554
				Train	52.8% (50.5%-55.0%)	0.18	48.0%	< 0.001 ***
Success after 3 years	OC	RF	60/60%	Test	49.4% (46.2%-52.6%)	0.07	48.1%	0.209
				Train	62.5% (60.0%-65.1%)	0.33	47.9%	< 0.001 ***
			70/30%	Test	48.6% (44.9%-52.3%)	0.06	48.1%	0.397
				Train	62.7% (60.3%-65.0%)	0.33	48.0%	< 0.001 ***
			80/20%	Test	48.5% (44.0%-53.1%)	0.04	48.1%	0.445
				Train	61.5% (59.3%-63.7%)	0.30	48.0%	< 0.001 ***
Success after 3 years	OC	PMR	60/60%	Test	48.8% (45.6%-52.0%)	0.09	48.1%	0.337
				Train	50.4% (47.8%-53.0%)	0.12	47.9%	0.034 *
			70/30%	Test	51.1% (47.4%-54.8%)	0.13	48.1%	0.054 .
				Train	50.4% (47.9%-52.8%)	0.12	48.0%	0.027 *
			80/20%	Test	49.8% (45.2%-54.4%)	0.10	48.1%	0.246
				Train	51.2% (48.9%-53.5%)	0.13	48.0%	0.002 **
Success after 3 years	OC	SGB	60/60%	Test	48.2% (45.0%-51.4%)	0.00	48.1%	0.487
				Train	48.0% (45.4%-50.6%)	0.00	47.9%	0.489
			70/30%	Test	48.3% (44.6%-52.1%)	0.01	48.1%	0.455
				Train	48.1% (45.7%-50.5%)	0.00	48.0%	0.471
			80/20%	Test	48.3% (43.8%-52.9%)	0.01	48.1%	0.482
				Train	48.3% (46.0%-50.5%)	0.01	48.0%	0.401
Success after 3 years	OC	BCART	60/60%	Test	43.3% (40.1%-46.5%)	0.08	48.1%	0.999
				Train	99.8% (99.4%-100.0%)	1.00	47.9%	< 0.001 ***
			70/30%	Test	42.5% (38.8%-46.2%)	0.06	48.1%	0.999
				Train	99.6% (99.1%-99.8%)	0.99	48.0%	< 0.001 ***
			80/20%	Test	43.5% (39.0%-48.1%)	0.08	48.1%	0.980
				Train	99.7% (99.4%-99.9%)	1.00	48.0%	< 0.001 ***
Success after 3 years	NC	CART	60/60%	Test	42.4% (41.4%-43.4%)	0.13	37.3%	< 0.001 ***
				Train	42.7% (41.9%-43.5%)	0.14	37.3%	< 0.001 ***
			70/30%	Test	42.7% (41.5%-43.8%)	0.15	37.3%	< 0.001 ***
				Train	43.1% (42.3%-43.8%)	0.15	37.3%	< 0.001 ***
			80/20%	Test	43.1% (41.7%-44.5%)	0.15	37.3%	< 0.001 ***
				Train	42.9% (42.2%-43.6%)	0.15	37.3%	< 0.001 ***

continued on next page

Table S1 – *continued from previous page*

Outcome	Group	Model	Split	Set	Accuracy	κ	NIR	p.value
Success after 3 years	NC	RF	60/60%	Test	47.0% (46.0%-48.0%)	0.20	37.3%	< 0.001 ***
				Train	60.8% (60.0%-61.6%)	0.41	37.3%	< 0.001 ***
			70/30%	Test	46.5% (45.4%-47.7%)	0.19	37.3%	< 0.001 ***
				Train	60.0% (59.3%-60.8%)	0.39	37.3%	< 0.001 ***
			80/20%	Test	46.0% (44.6%-47.4%)	0.18	37.3%	< 0.001 ***
				Train	59.4% (58.7%-60.1%)	0.38	37.3%	< 0.001 ***
Success after 3 years	NC	PMR	60/60%	Test	45.8% (44.8%-46.8%)	0.18	37.3%	< 0.001 ***
				Train	45.9% (45.1%-46.8%)	0.18	37.3%	< 0.001 ***
			70/30%	Test	46.4% (45.2%-47.5%)	0.19	37.3%	< 0.001 ***
				Train	45.3% (44.6%-46.1%)	0.17	37.3%	< 0.001 ***
			80/20%	Test	46.5% (45.1%-47.9%)	0.19	37.3%	< 0.001 ***
				Train	45.7% (45.0%-46.4%)	0.18	37.3%	< 0.001 ***
Success after 3 years	NC	SGB	60/60%	Test	37.7% (36.7%-38.7%)	0.01	37.3%	0.228
				Train	37.8% (37.0%-38.6%)	0.01	37.3%	0.142
			70/30%	Test	37.7% (36.6%-38.8%)	0.01	37.3%	0.284
				Train	37.7% (37.0%-38.5%)	0.01	37.3%	0.138
			80/20%	Test	37.7% (36.3%-39.1%)	0.01	37.3%	0.312
				Train	37.7% (37.0%-38.4%)	0.01	37.3%	0.151
Success after 3 years	NC	BCART	60/60%	Test	39.6% (38.6%-40.6%)	0.09	37.3%	< 0.001 ***
				Train	98.5% (98.3%-98.7%)	0.98	37.3%	< 0.001 ***
			70/30%	Test	42.1% (40.9%-43.2%)	0.13	37.3%	< 0.001 ***
				Train	98.2% (98.0%-98.4%)	0.97	37.3%	< 0.001 ***
			80/20%	Test	40.7% (39.3%-42.1%)	0.11	37.3%	< 0.001 ***
				Train	98.2% (98.0%-98.4%)	0.97	37.3%	< 0.001 ***

NIR, No information rate; AS, students with ASD; OC, students with other conditions; NC, students with no recorded conditions; CART, classification and regression trees; RF, random forest; PMR, penalized multinomial regression; SGB, stochastic gradient boosting; BCART, bagged CART; . = $p < 0.1$, * = $p < 0.05$, ** = $p < 0.01$, *** = $p < 0.001$

Table S2

Model performance for the three participant groups SAMPLED

Outcome	Group	Model	Split	Set	Accuracy	κ	NIR	p.value
Dropout after 1 year	AS	CART	60/40%	Test	76.3% (59.8%-88.6%)	0.00	76.3%	0.588
				Train	76.3% (63.4%-86.4%)	0.00	76.3%	0.571
			70/30%	Test	78.6% (59.0%-91.7%)	0.00	78.6%	0.607
				Train	75.4% (63.5%-84.9%)	0.00	75.4%	0.565
			80/20%	Test	77.8% (52.4%-93.6%)	0.00	77.8%	0.630
				Train	75.9% (65.0%-84.9%)	0.00	75.9%	0.561
Dropout after 1 year	AS	RF	60/40%	Test	76.3% (59.8%-88.6%)	0.00	76.3%	0.588
				Train	79.7% (67.2%-89.0%)	0.20	76.3%	0.331
			70/30%	Test	78.6% (59.0%-91.7%)	0.00	78.6%	0.607
				Train	82.6% (71.6%-90.7%)	0.39	75.4%	0.101
			80/20%	Test	77.8% (52.4%-93.6%)	0.00	77.8%	0.630
				Train	79.7% (69.2%-88.0%)	0.22	75.9%	0.260
Dropout after 1 year	AS	PMR	60/40%	Test	73.7% (56.9%-86.6%)	-0.05	76.3%	0.724
				Train	83.1% (71.0%-91.6%)	0.38	76.3%	0.141
			70/30%	Test	71.4% (51.3%-86.8%)	0.15	78.6%	0.873
				Train	79.7% (68.3%-88.4%)	0.28	75.4%	0.246
			80/20%	Test	83.3% (58.6%-96.4%)	0.34	77.8%	0.409
				Train	77.2% (66.4%-85.9%)	0.12	75.9%	0.457
Dropout after 1 year	AS	SGB	60/40%	Test	73.7% (56.9%-86.6%)	-0.05	76.3%	0.724
				Train	81.4% (69.1%-90.3%)	0.29	76.3%	0.226
			70/30%	Test	75.0% (55.1%-89.3%)	-0.07	78.6%	0.762
				Train	78.3% (66.7%-87.3%)	0.21	75.4%	0.345
			80/20%	Test	66.7% (41.0%-86.7%)	-0.17	77.8%	0.916
				Train	77.2% (66.4%-85.9%)	0.08	75.9%	0.457
Dropout after 1 year	AS	BCART	60/40%	Test	73.7% (56.9%-86.6%)	0.14	76.3%	0.724
				Train	98.3% (90.9%-100.0%)	0.95	76.3%	< 0.001 ***
			70/30%	Test	82.1% (63.1%-93.9%)	0.24	78.6%	0.426
				Train	100.0% (94.8%-100.0%)	1.00	75.4%	< 0.001 ***
			80/20%	Test	66.7% (41.0%-86.7%)	-0.17	77.8%	0.916
				Train	98.7% (93.1%-100.0%)	0.96	75.9%	< 0.001 ***
Dropout after 1 year	OC	CART	60/40%	Test	78.9% (62.7%-90.4%)	0.00	78.9%	0.593
				Train	78.0% (65.3%-87.7%)	0.00	78.0%	0.574
			70/30%	Test	78.6% (59.0%-91.7%)	0.00	78.6%	0.607
				Train	78.3% (66.7%-87.3%)	0.00	78.3%	0.569
			80/20%	Test	78.9% (54.4%-93.9%)	0.00	78.9%	0.630
				Train	78.2% (67.4%-86.8%)	0.00	78.2%	0.565
Dropout after 1 year	OC	RF	60/40%	Test	78.9% (62.7%-90.4%)	0.00	78.9%	0.593
				Train	79.7% (67.2%-89.0%)	0.12	78.0%	0.449
			70/30%	Test	78.6% (59.0%-91.7%)	0.00	78.6%	0.607

continued on next page

Table S2 – *continued from previous page*

Outcome	Group	Model	Split	Set	Accuracy	κ	NIR	p.value
				Train	79.7% (68.3%-88.4%)	0.10	78.3%	0.453
			80/20%	Test	78.9% (54.4%-93.9%)	0.00	78.9%	0.630
				Train	78.2% (67.4%-86.8%)	0.00	78.2%	0.565
Dropout after 1 year	OC	PMR	60/40%	Test	78.9% (62.7%-90.4%)	0.00	78.9%	0.593
				Train	78.0% (65.3%-87.7%)	0.00	78.0%	0.574
			70/30%	Test	78.6% (59.0%-91.7%)	0.00	78.6%	0.607
				Train	78.3% (66.7%-87.3%)	0.00	78.3%	0.569
			80/20%	Test	78.9% (54.4%-93.9%)	0.00	78.9%	0.630
				Train	78.2% (67.4%-86.8%)	0.00	78.2%	0.565
Dropout after 1 year	OC	SGB	60/40%	Test	71.1% (54.1%-84.6%)	0.09	78.9%	0.914
				Train	79.7% (67.2%-89.0%)	0.18	78.0%	0.449
			70/30%	Test	64.3% (44.1%-81.4%)	-0.06	78.6%	0.976
				Train	82.6% (71.6%-90.7%)	0.37	78.3%	0.237
			80/20%	Test	78.9% (54.4%-93.9%)	0.00	78.9%	0.630
				Train	78.2% (67.4%-86.8%)	0.00	78.2%	0.565
Dropout after 1 year	OC	BCART	60/40%	Test	71.1% (54.1%-84.6%)	-0.01	78.9%	0.914
				Train	98.3% (90.9%-100.0%)	0.95	78.0%	< 0.001 ***
			70/30%	Test	46.4% (27.5%-66.1%)	-0.22	78.6%	1.000
				Train	98.6% (92.2%-100.0%)	0.96	78.3%	< 0.001 ***
			80/20%	Test	68.4% (43.4%-87.4%)	-0.16	78.9%	0.915
				Train	100.0% (95.4%-100.0%)	1.00	78.2%	< 0.001 ***
Dropout after 1 year	NC	CART	60/40%	Test	55.3% (38.3%-71.4%)	-0.05	73.7%	0.996
				Train	74.6% (61.6%-85.0%)	0.35	71.2%	0.339
			70/30%	Test	62.1% (42.3%-79.3%)	0.09	72.4%	0.923
				Train	76.5% (64.6%-85.9%)	0.40	72.1%	0.253
			80/20%	Test	73.7% (48.8%-90.9%)	0.00	73.7%	0.617
				Train	71.8% (60.5%-81.4%)	0.00	71.8%	0.557
Dropout after 1 year	NC	RF	60/40%	Test	73.7% (56.9%-86.6%)	0.00	73.7%	0.584
				Train	72.9% (59.7%-83.6%)	0.08	71.2%	0.451
			70/30%	Test	72.4% (52.8%-87.3%)	0.00	72.4%	0.594
				Train	76.5% (64.6%-85.9%)	0.21	72.1%	0.253
			80/20%	Test	73.7% (48.8%-90.9%)	0.00	73.7%	0.617
				Train	76.9% (66.0%-85.7%)	0.24	71.8%	0.190
Dropout after 1 year	NC	PMR	60/40%	Test	71.1% (54.1%-84.6%)	-0.05	73.7%	0.716
				Train	72.9% (59.7%-83.6%)	0.08	71.2%	0.451
			70/30%	Test	69.0% (49.2%-84.7%)	-0.07	72.4%	0.739
				Train	75.0% (63.0%-84.7%)	0.14	72.1%	0.349
			80/20%	Test	73.7% (48.8%-90.9%)	0.00	73.7%	0.617
				Train	74.4% (63.2%-83.6%)	0.13	71.8%	0.359
Dropout after 1 year	NC	SGB	60/40%	Test	71.1% (54.1%-84.6%)	0.23	73.7%	0.716
				Train	79.7% (67.2%-89.0%)	0.47	71.2%	0.095 .
			70/30%	Test	55.2% (35.7%-73.6%)	-0.17	72.4%	0.986

continued on next page

Table S2 – *continued from previous page*

Outcome	Group	Model	Split	Set	Accuracy	κ	NIR	p.value
				Train	77.9% (66.2%-87.1%)	0.38	72.1%	0.173
			80/20%	Test	73.7% (48.8%-90.9%)	0.00	73.7%	0.617
				Train	76.9% (66.0%-85.7%)	0.24	71.8%	0.190
Dropout after 1 year	NC	BCART	60/40%	Test	52.6% (35.8%-69.0%)	-0.15	73.7%	0.998
				Train	98.3% (90.9%-100.0%)	0.96	71.2%	< 0.001 ***
			70/30%	Test	51.7% (32.5%-70.6%)	-0.12	72.4%	0.995
				Train	100.0% (94.7%-100.0%)	1.00	72.1%	< 0.001 ***
			80/20%	Test	73.7% (48.8%-90.9%)	0.00	73.7%	0.617
				Train	100.0% (95.4%-100.0%)	1.00	71.8%	< 0.001 ***
Dropout after 2 years	AS	CART	60/40%	Test	50.0% (33.4%-66.6%)	-0.12	63.2%	0.966
				Train	72.9% (59.7%-83.6%)	0.39	62.7%	0.067 .
			70/30%	Test	67.9% (47.6%-84.1%)	0.17	64.3%	0.429
				Train	73.9% (61.9%-83.7%)	0.37	62.3%	0.029 *
			80/20%	Test	63.2% (38.4%-83.7%)	0.00	63.2%	0.601
				Train	62.8% (51.1%-73.5%)	0.00	62.8%	0.551
Dropout after 2 years	AS	RF	60/40%	Test	73.7% (56.9%-86.6%)	0.36	63.2%	0.118
				Train	72.9% (59.7%-83.6%)	0.32	62.7%	0.067 .
			70/30%	Test	75.0% (55.1%-89.3%)	0.39	64.3%	0.162
				Train	73.9% (61.9%-83.7%)	0.36	62.3%	0.029 *
			80/20%	Test	63.2% (38.4%-83.7%)	0.13	63.2%	0.601
				Train	83.3% (73.2%-90.8%)	0.61	62.8%	< 0.001 ***
Dropout after 2 years	AS	PMR	60/40%	Test	73.7% (56.9%-86.6%)	0.38	63.2%	0.118
				Train	72.9% (59.7%-83.6%)	0.32	62.7%	0.067 .
			70/30%	Test	53.6% (33.9%-72.5%)	0.05	64.3%	0.914
				Train	76.8% (65.1%-86.1%)	0.47	62.3%	0.008 **
			80/20%	Test	63.2% (38.4%-83.7%)	0.13	63.2%	0.601
				Train	76.9% (66.0%-85.7%)	0.43	62.8%	0.006 **
Dropout after 2 years	AS	SGB	60/40%	Test	57.9% (40.8%-73.7%)	0.01	63.2%	0.801
				Train	76.3% (63.4%-86.4%)	0.44	62.7%	0.019 *
			70/30%	Test	64.3% (44.1%-81.4%)	0.15	64.3%	0.585
				Train	76.8% (65.1%-86.1%)	0.48	62.3%	0.008 **
			80/20%	Test	68.4% (43.4%-87.4%)	0.23	63.2%	0.414
				Train	70.5% (59.1%-80.3%)	0.27	62.8%	0.097 .
Dropout after 2 years	AS	BCART	60/40%	Test	57.9% (40.8%-73.7%)	0.04	63.2%	0.801
				Train	100.0% (93.9%-100.0%)	1.00	62.7%	< 0.001 ***
			70/30%	Test	50.0% (30.6%-69.4%)	-0.14	64.3%	0.960
				Train	100.0% (94.8%-100.0%)	1.00	62.3%	< 0.001 ***
			80/20%	Test	63.2% (38.4%-83.7%)	0.13	63.2%	0.601
				Train	100.0% (95.4%-100.0%)	1.00	62.8%	< 0.001 ***
Dropout after 2 years	OC	CART	60/40%	Test	76.3% (59.8%-88.6%)	0.00	76.3%	0.588
				Train	74.6% (61.6%-85.0%)	0.00	74.6%	0.569

continued on next page

Table S2 – *continued from previous page*

Outcome	Group	Model	Split	Set	Accuracy	κ	NIR	p.value
			70/30%	Test	75.0% (55.1%-89.3%)	0.00	75.0%	0.600
				Train	75.4% (63.5%-84.9%)	0.00	75.4%	0.565
			80/20%	Test	77.8% (52.4%-93.6%)	0.00	77.8%	0.630
				Train	74.7% (63.6%-83.8%)	0.00	74.7%	0.560
Dropout after 2 years	OC	RF	60/40%	Test	76.3% (59.8%-88.6%)	0.00	76.3%	0.588
				Train	74.6% (61.6%-85.0%)	0.00	74.6%	0.569
			70/30%	Test	75.0% (55.1%-89.3%)	0.00	75.0%	0.600
				Train	75.4% (63.5%-84.9%)	0.00	75.4%	0.565
			80/20%	Test	77.8% (52.4%-93.6%)	0.00	77.8%	0.630
				Train	75.9% (65.0%-84.9%)	0.07	74.7%	0.457
Dropout after 2 years	OC	PMR	60/40%	Test	76.3% (59.8%-88.6%)	0.00	76.3%	0.588
				Train	74.6% (61.6%-85.0%)	0.00	74.6%	0.569
			70/30%	Test	75.0% (55.1%-89.3%)	0.00	75.0%	0.600
				Train	76.8% (65.1%-86.1%)	0.09	75.4%	0.454
			80/20%	Test	77.8% (52.4%-93.6%)	0.00	77.8%	0.630
				Train	75.9% (65.0%-84.9%)	0.07	74.7%	0.457
Dropout after 2 years	OC	SGB	60/40%	Test	73.7% (56.9%-86.6%)	-0.05	76.3%	0.724
				Train	76.3% (63.4%-86.4%)	0.10	74.6%	0.450
			70/30%	Test	75.0% (55.1%-89.3%)	0.00	75.0%	0.600
				Train	76.8% (65.1%-86.1%)	0.14	75.4%	0.454
			80/20%	Test	77.8% (52.4%-93.6%)	0.00	77.8%	0.630
				Train	74.7% (63.6%-83.8%)	0.00	74.7%	0.560
Dropout after 2 years	OC	BCART	60/40%	Test	68.4% (51.3%-82.5%)	-0.03	76.3%	0.906
				Train	100.0% (93.9%-100.0%)	1.00	74.6%	< 0.001 ***
			70/30%	Test	64.3% (44.1%-81.4%)	-0.18	75.0%	0.932
				Train	100.0% (94.8%-100.0%)	1.00	75.4%	< 0.001 ***
			80/20%	Test	66.7% (41.0%-86.7%)	-0.17	77.8%	0.916
				Train	100.0% (95.4%-100.0%)	1.00	74.7%	< 0.001 ***
Dropout after 2 years	NC	CART	60/40%	Test	71.1% (54.1%-84.6%)	0.00	71.1%	0.580
				Train	69.5% (56.1%-80.8%)	0.00	69.5%	0.563
			70/30%	Test	64.3% (44.1%-81.4%)	-0.03	71.4%	0.852
				Train	78.3% (66.7%-87.3%)	0.38	69.6%	0.072 .
			80/20%	Test	50.0% (26.0%-74.0%)	-0.05	72.2%	0.988
				Train	77.2% (66.4%-85.9%)	0.43	69.6%	0.087 .
Dropout after 2 years	NC	RF	60/40%	Test	71.1% (54.1%-84.6%)	0.00	71.1%	0.580
				Train	76.3% (63.4%-86.4%)	0.28	69.5%	0.161
			70/30%	Test	71.4% (51.3%-86.8%)	0.00	71.4%	0.594
				Train	76.8% (65.1%-86.1%)	0.30	69.6%	0.118
			80/20%	Test	66.7% (41.0%-86.7%)	-0.10	72.2%	0.789
				Train	75.9% (65.0%-84.9%)	0.27	69.6%	0.135
Dropout after 2 years	NC	PMR	60/40%	Test	71.1% (54.1%-84.6%)	0.00	71.1%	0.580
				Train	71.2% (57.9%-82.2%)	0.08	69.5%	0.451

continued on next page

Table S2 – *continued from previous page*

Outcome	Group	Model	Split	Set	Accuracy	κ	NIR	p.value
			70/30%	Test	71.4% (51.3%-86.8%)	0.00	71.4%	0.594
				Train	71.0% (58.8%-81.3%)	0.07	69.6%	0.455
			80/20%	Test	66.7% (41.0%-86.7%)	-0.10	72.2%	0.789
				Train	70.9% (59.6%-80.6%)	0.09	69.6%	0.458
Dropout after 2 years	NC	SGB	60/40%	Test	65.8% (48.6%-80.4%)	-0.10	71.1%	0.816
				Train	71.2% (57.9%-82.2%)	0.18	69.5%	0.451
			70/30%	Test	67.9% (47.6%-84.1%)	0.11	71.4%	0.741
				Train	75.4% (63.5%-84.9%)	0.39	69.6%	0.181
			80/20%	Test	66.7% (41.0%-86.7%)	0.17	72.2%	0.789
				Train	88.6% (79.5%-94.7%)	0.71	69.6%	< 0.001 ***
Dropout after 2 years	NC	BCART	60/40%	Test	65.8% (48.6%-80.4%)	0.14	71.1%	0.816
				Train	100.0% (93.9%-100.0%)	1.00	69.5%	< 0.001 ***
			70/30%	Test	60.7% (40.6%-78.5%)	0.00	71.4%	0.925
				Train	100.0% (94.8%-100.0%)	1.00	69.6%	< 0.001 ***
			80/20%	Test	66.7% (41.0%-86.7%)	0.26	72.2%	0.789
				Train	100.0% (95.4%-100.0%)	1.00	69.6%	< 0.001 ***
Success after 3 years	AS	CART	60/40%	Test	39.5% (24.0%-56.6%)	0.05	42.1%	0.686
				Train	61.0% (47.4%-73.5%)	0.40	42.4%	0.003 **
			70/30%	Test	42.9% (24.5%-62.8%)	0.00	42.9%	0.572
				Train	42.0% (30.2%-54.5%)	0.00	42.0%	0.546
			80/20%	Test	42.1% (20.3%-66.5%)	0.00	42.1%	0.587
				Train	42.3% (31.2%-54.0%)	0.00	42.3%	0.543
Success after 3 years	AS	RF	60/40%	Test	50.0% (33.4%-66.6%)	0.15	42.1%	0.205
				Train	66.1% (52.6%-77.9%)	0.44	42.4%	< 0.001 ***
			70/30%	Test	64.3% (44.1%-81.4%)	0.40	42.9%	0.018 *
				Train	68.1% (55.8%-78.8%)	0.47	42.0%	< 0.001 ***
			80/20%	Test	63.2% (38.4%-83.7%)	0.38	42.1%	0.053 .
				Train	67.9% (56.4%-78.1%)	0.47	42.3%	< 0.001 ***
Success after 3 years	AS	PMR	60/40%	Test	36.8% (21.8%-54.0%)	0.04	42.1%	0.793
				Train	69.5% (56.1%-80.8%)	0.52	42.4%	< 0.001 ***
			70/30%	Test	57.1% (37.2%-75.5%)	0.27	42.9%	0.091 .
				Train	59.4% (46.9%-71.1%)	0.32	42.0%	0.003 **
			80/20%	Test	52.6% (28.9%-75.6%)	0.20	42.1%	0.241
				Train	59.0% (47.3%-70.0%)	0.31	42.3%	0.002 **
Success after 3 years	AS	SGB	60/40%	Test	42.1% (26.3%-59.2%)	0.00	42.1%	0.562
				Train	42.4% (29.6%-55.9%)	0.01	42.4%	0.550
			70/30%	Test	46.4% (27.5%-66.1%)	0.07	42.9%	0.421
				Train	44.9% (32.9%-57.4%)	0.05	42.0%	0.355
			80/20%	Test	47.4% (24.4%-71.1%)	0.10	42.1%	0.404
				Train	42.3% (31.2%-54.0%)	0.00	42.3%	0.543
Success after 3 years	AS	BCART	60/40%	Test	34.2% (19.6%-51.4%)	-0.04	42.1%	0.876

continued on next page

Table S2 – *continued from previous page*

Outcome	Group	Model	Split	Set	Accuracy	κ	NIR	p.value
				Train	100.0% (93.9%-100.0%)	1.00	42.4%	< 0.001 ***
			70/30%	Test	28.6% (13.2%-48.7%)	-0.15	42.9%	0.960
				Train	100.0% (94.8%-100.0%)	1.00	42.0%	< 0.001 ***
			80/20%	Test	15.8% (3.4%-39.6%)	-0.33	42.1%	0.997
				Train	100.0% (95.4%-100.0%)	1.00	42.3%	< 0.001 ***
Success after 3 years	OC	CART	60/40%	Test	52.6% (35.8%-69.0%)	0.00	52.6%	0.565
				Train	50.8% (37.5%-64.1%)	0.00	50.8%	0.552
			70/30%	Test	53.6% (33.9%-72.5%)	0.00	53.6%	0.577
				Train	50.7% (38.4%-63.0%)	0.00	50.7%	0.548
			80/20%	Test	55.6% (30.8%-78.5%)	0.00	55.6%	0.597
				Train	50.6% (39.1%-62.1%)	0.00	50.6%	0.545
Success after 3 years	OC	RF	60/40%	Test	52.6% (35.8%-69.0%)	0.00	52.6%	0.565
				Train	52.5% (39.1%-65.7%)	0.04	50.8%	0.449
			70/30%	Test	53.6% (33.9%-72.5%)	0.00	53.6%	0.577
				Train	55.1% (42.6%-67.1%)	0.11	50.7%	0.274
			80/20%	Test	55.6% (30.8%-78.5%)	0.00	55.6%	0.597
				Train	51.9% (40.4%-63.3%)	0.03	50.6%	0.455
Success after 3 years	OC	PMR	60/40%	Test	52.6% (35.8%-69.0%)	0.03	52.6%	0.565
				Train	54.2% (40.8%-67.3%)	0.09	50.8%	0.348
			70/30%	Test	46.4% (27.5%-66.1%)	-0.10	53.6%	0.828
				Train	56.5% (44.0%-68.4%)	0.14	50.7%	0.200
			80/20%	Test	55.6% (30.8%-78.5%)	0.00	55.6%	0.597
				Train	51.9% (40.4%-63.3%)	0.03	50.6%	0.455
Success after 3 years	OC	SGB	60/40%	Test	57.9% (40.8%-73.7%)	0.22	52.6%	0.314
				Train	62.7% (49.1%-75.0%)	0.38	50.8%	0.045 *
			70/30%	Test	53.6% (33.9%-72.5%)	0.16	53.6%	0.577
				Train	63.8% (51.3%-75.0%)	0.40	50.7%	0.020 *
			80/20%	Test	33.3% (13.3%-59.0%)	-0.09	55.6%	0.984
				Train	67.1% (55.6%-77.3%)	0.46	50.6%	0.002 **
Success after 3 years	OC	BCART	60/40%	Test	36.8% (21.8%-54.0%)	-0.07	52.6%	0.983
				Train	100.0% (93.9%-100.0%)	1.00	50.8%	< 0.001 ***
			70/30%	Test	46.4% (27.5%-66.1%)	0.03	53.6%	0.828
				Train	100.0% (94.8%-100.0%)	1.00	50.7%	< 0.001 ***
			80/20%	Test	44.4% (21.5%-69.2%)	0.00	55.6%	0.882
				Train	100.0% (95.4%-100.0%)	1.00	50.6%	< 0.001 ***
Success after 3 years	NC	CART	60/40%	Test	31.6% (17.5%-48.7%)	-0.02	39.5%	0.878
				Train	55.9% (42.4%-68.8%)	0.35	40.7%	0.013 *
			70/30%	Test	39.3% (21.5%-59.4%)	0.00	39.3%	0.571
				Train	40.6% (28.9%-53.1%)	0.00	40.6%	0.546
			80/20%	Test	38.9% (17.3%-64.3%)	0.07	38.9%	0.588
				Train	59.5% (47.9%-70.4%)	0.37	40.5%	0.001 ***
Success after 3 years	NC	RF	60/40%	Test	39.5% (24.0%-56.6%)	0.05	39.5%	0.561

continued on next page

Table S2 – *continued from previous page*

Outcome	Group	Model	Split	Set	Accuracy	κ	NIR	p.value
				Train	98.3% (90.9%-100.0%)	0.97	40.7%	< 0.001 ***
			70/30%	Test	50.0% (30.6%-69.4%)	0.23	39.3%	0.166
				Train	100.0% (94.8%-100.0%)	1.00	40.6%	< 0.001 ***
			80/20%	Test	38.9% (17.3%-64.3%)	0.06	38.9%	0.588
				Train	98.7% (93.1%-100.0%)	0.98	40.5%	< 0.001 ***
Success after 3 years	NC	PMR	60/40%	Test	26.3% (13.4%-43.1%)	-0.12	39.5%	0.969
				Train	55.9% (42.4%-68.8%)	0.33	40.7%	0.013 *
			70/30%	Test	39.3% (21.5%-59.4%)	0.06	39.3%	0.571
				Train	62.3% (49.8%-73.7%)	0.42	40.6%	< 0.001 ***
			80/20%	Test	44.4% (21.5%-69.2%)	0.15	38.9%	0.398
				Train	54.4% (42.8%-65.7%)	0.30	40.5%	0.009 **
Success after 3 years	NC	SGB	60/40%	Test	34.2% (19.6%-51.4%)	-0.01	39.5%	0.795
				Train	76.3% (63.4%-86.4%)	0.63	40.7%	< 0.001 ***
			70/30%	Test	28.6% (13.2%-48.7%)	-0.10	39.3%	0.915
				Train	68.1% (55.8%-78.8%)	0.51	40.6%	< 0.001 ***
			80/20%	Test	27.8% (9.7%-53.5%)	-0.12	38.9%	0.889
				Train	73.4% (62.3%-82.7%)	0.59	40.5%	< 0.001 ***
Success after 3 years	NC	BCART	60/40%	Test	50.0% (33.4%-66.6%)	0.24	39.5%	0.123
				Train	98.3% (90.9%-100.0%)	0.97	40.7%	< 0.001 ***
			70/30%	Test	50.0% (30.6%-69.4%)	0.23	39.3%	0.166
				Train	100.0% (94.8%-100.0%)	1.00	40.6%	< 0.001 ***
			80/20%	Test	33.3% (13.3%-59.0%)	-0.04	38.9%	0.763
				Train	100.0% (95.4%-100.0%)	1.00	40.5%	< 0.001 ***

NIR, No information rate; AS, students with ASD; OC, students with other conditions; NC, students with no recorded conditions; CART, classification and regression trees; RF, random forest; PMR, penalized multinomial regression; SGB, stochastic gradient boosting; BCART, bagged CART; . = p < 0.1, * = p < 0.05, ** = p < 0.01, *** = p < 0.001
